# Non-random patterns in viral diversity

**DOI:** 10.1038/ncomms9147

**Published:** 2015-09-22

**Authors:** Simon J. Anthony, Ariful Islam, Christine Johnson, Isamara Navarrete-Macias, Eliza Liang, Komal Jain, Peta L. Hitchens, Xiaoyu Che, Alexander Soloyvov, Allison L. Hicks, Rafael Ojeda-Flores, Carlos Zambrana-Torrelio, Werner Ulrich, Melinda K. Rostal, Alexandra Petrosov, Joel Garcia, Najmul Haider, Nathan Wolfe, Tracey Goldstein, Stephen S. Morse, Mahmudur Rahman, Jonathan H. Epstein, Jonna K. Mazet, Peter Daszak, W. Ian Lipkin

**Affiliations:** 1 Center for Infection and Immunity, Mailman School of Public Health, Columbia University, 722 West 168th Street, New York, New York 10032, USA; 2 Department of Epidemiology, Mailman School of Public Health, Columbia University, 722 West 168th Street, New York, New York 10032, USA; 3 EcoHealth Alliance, 460 West 34th Street, New York, New York 10001, USA; 4 One Health Institute & Wildlife Health Center, School of Veterinary Medicine, University of California, Davis, California 95616, USA; 5 Department of Animal Environment and Health, Swedish University of Agricultural Sciences, PO Box 7068, SE-750 07 Uppsala, Sweden; 6 Facultad de Medicina Veterinaria y Zootecnia, Universidad Nacional Autónoma de México, Ciudad Universitaria, México D.F. 04510, Mexico; 7 Nicolaus Copernicus University, Lwowska 1, 87-100 Toruń, Poland; 8 International Centre for Diahorreal Disease Research, Bangladesh (icddr,b), GPO Box 128, Dhaka 1000, Bangladesh; 9 Section for Epidemiology, National Veterinary Institute, Technical University of Denmark, Bülowsvej 27, DK-1870 Frederiksberg C, Denmark; 10 Metabiota, Inc. One Sutter, Suite 600, San Francisco, CA 94104, USA; 11 IEDCR (Institute of epidemiology and disease control research), Ministry of Health and Family Welfare, Government of Bangladesh, Mohakhali, Dhaka 1212, Bangladesh

## Abstract

It is currently unclear whether changes in viral communities will ever be predictable. Here we investigate whether viral communities in wildlife are inherently structured (inferring predictability) by looking at whether communities are assembled through deterministic (often predictable) or stochastic (not predictable) processes. We sample macaque faeces across nine sites in Bangladesh and use consensus PCR and sequencing to discover 184 viruses from 14 viral families. We then use network modelling and statistical null-hypothesis testing to show the presence of non-random deterministic patterns at different scales, between sites and within individuals. We show that the effects of determinism are not absolute however, as stochastic patterns are also observed. In showing that determinism is an important process in viral community assembly we conclude that it should be possible to forecast changes to some portion of a viral community, however there will always be some portion for which prediction will be unlikely.

## Abstract (web summary)

It is unclear whether the emergence of new infectious diseases can be predicted. Here, Anthony *et al*. investigate viral communities in faeces of wild macaques and show that viral diversity is inherently structured, suggesting that it should be possible to forecast some changes in viral communities.

## Graphical abstract

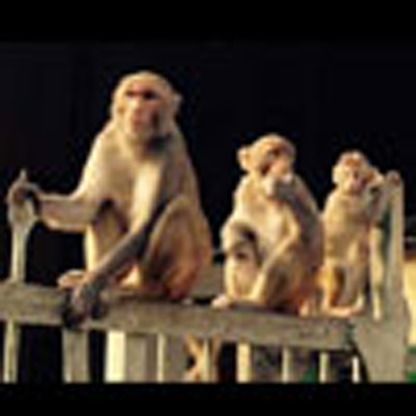

The recent Ebola virus outbreak in West Africa^1^,^2^ is a timely reminder that we have never successfully predicted the emergence of a new infectious disease in people^3^. Perhaps precluded from doing so (at least in part) by historical deficiencies in our knowledge of global viral diversity in wildlife^4^,^5^,^6^ or the pathways and mechanisms of spillover and spread^7^,^8^,^9^, the threat that infectious diseases now increasingly pose to public health^4^,^5^,^6^ and economic stability^10^,^11^,^12^ has excited efforts to establish a predictive understanding of emergence^3^,^4^,^13^. One area of ‘prediction' that would be particularly useful is the ability to forecast how viral diversity might respond to environmental drivers of disease emergence, for example land-use change. This would allow us to test response options designed to mitigate, or adapt to, the impact of those changes and potentially reduce the risk of zoonotic emergence^14^. Being able to predict such changes however assumes that viral diversity is inherently predictable. It assumes that viral communities are built and controlled through deterministic and inherently ecological processes that can be identified and understood, and we do not yet know whether this is true. If viral diversity in wildlife is inherently random (stochastic), then predicting the outcome of an environmental perturbation would be impossible, as many have long believed^15^. But if it is not random, if it is deterministically structured (or at least structured to some degree) then predicting changes in viral diversity might indeed be possible.

Here, we apply established ecological theory on macrobial species distributions (for example, plants and animals)^16^,^17^ to viral assemblages of the rhesus macaque and look for evidence of deterministic and stochastic effects in the structure of these communities. We adopt the null hypothesis that virodiversity can be readily explained by random processes (chance colonisation or extinction and ecological drift) and look for departure from random via the presence of discernible pattern to identify and subsequently test for the presence of determinism^18^,^19^. Our data indicate that viral communities within the macaque are assembled though largely ecological (deterministic) processes and should therefore be inherently predictable. However, we also show that stochastic processes contribute to patterns of viral diversity, suggesting that changes to some portion of the community will never be predictable.

Throughout the paper we use the terms ‘determinism' to refer to the identification of non-random patterns and ‘stochastic' to refer to any ecological process that results in patterns of diversity, relative abundance and composition that are indistinguishable from random chance alone^18^. We clarify that it is not our intention at this time to determine the processes behind non-randomness, as these might involve a variety of either neutral processes assuming ecological equivalence^17^ or processes based on ecological niche differentiation^16^.

## Results

### Virodiversity of the rhesus macaque in Bangladesh

**Figure 1:**
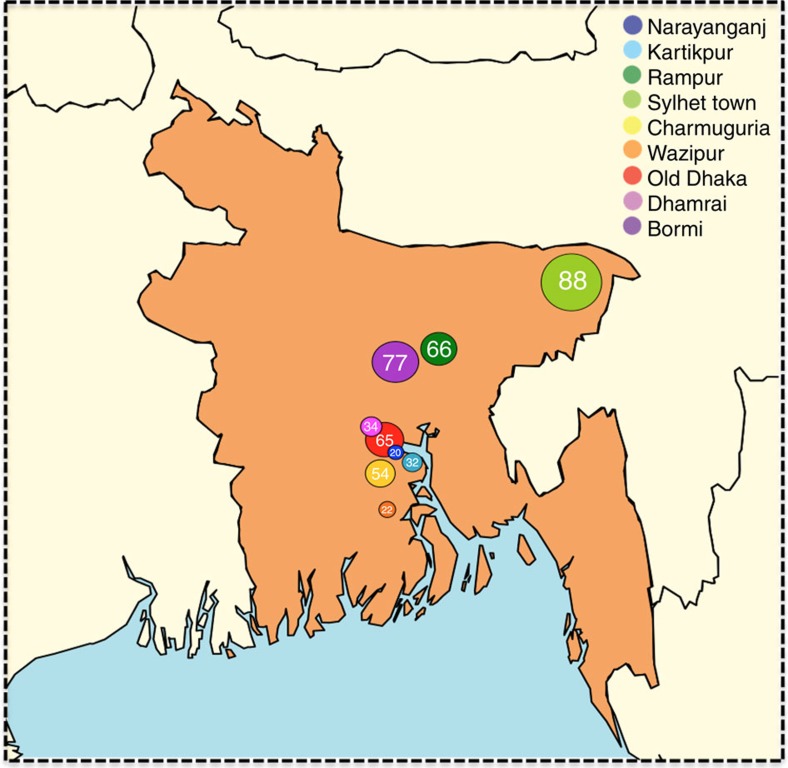
Distribution of the nine macaque sampling sites. Number of individuals sampled at each site is indicated. All sites are urban/peri-urban, with known contact between macaques and people, livestock and domestic animals (though frequency of contact is not assessed here). Number of samples collected is not consistent across sites, however sampling effort (number of collection days per site) is the same. Further description of each site is provided in Supplementary Table 3.

**Figure 2:**
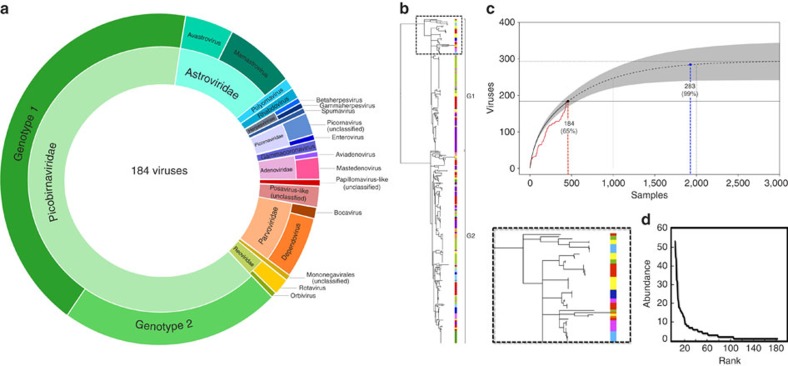
Virodiversity of rhesus macaques in Bangladesh. (**a**) Diversity of viruses discovered by family. (**b**) Maximum likelihood phylogeny of picobirnavirus diversity. Although viruses from many families were discovered, we have selected to show the PbV tree because of the substantial diversity observed. One section of the tree is shown as an insert for better visualization. (**c**) Viral discovery curve to assess saturation and estimate the total richnesss (number of viruses) that exists. Red line is the collector curve. Solid black line is the rarefaction curve. Dotted line indicates Chao2 estimation of asymptotic richness by sample number, and shading indicates 95% confidence intervals. (**d**) Rank abundance curve of the 184 viruses detected in this study.

Using a combination of consensus polymerase chain reaction (cPCR) and high-throughput sequencing (HTS), we characterised the faecal virodiversity of 458 rhesus macaques sampled across nine urban sites in Bangladesh (Fig. 1) and identified 184 unique viruses from 14 families (Fig. 2a and Supplementary Table 1). We identified 37/184 viruses by cPCR and 147/184 by HTS — highlighting the usefulness of combining the high sensitivity of PCR with the broad reactivity of HTS. We make particular note of an unprecedented diversity of a small bipartite picobirnaviruses (PbVs), which accounted for 120 of the 184 viruses found in these animals (Fig. 2b). Importantly, we make no assertion that all 184 viruses are singularly associated with macaques, or that true infection has occurred. Indeed, several human viruses were detected during this discovery effort (Supplementary Table 1) suggesting a multihost ecology that would be readily explained by the long and close association between people and macaques at each of our sampling sites^21^,^22^. Instead, we use genetic detection to demonstrate inclusion in the viral community to which these macaques are exposed, even if the presence of a virus is the result of dispersal from another host species or contribution to the community is low because of rarity^23^. For purposes of definition, we consider a ‘unique virus' to be a monophyletic cluster of sequences that is distinct from its nearest neighbour by non-overlapping genetic identities^24^.

Non-parametric viral discovery curves were used to assess the bounds of the viral community (total number of viruses) and assess the completeness of our discovery effort^24^,^25^,^26^. These curves indicated that the community contains a total of 283 viruses (Fig. 2c). We estimate therefore that the 184 viruses detected in our study represent ∼65% of the viruses that exist in these macaques. Plotting the rank abundance of the observed virodiversity showed that only a few of these viruses dominated the community, whereas most occurred only rarely (Fig. 2d). This uneven distribution is a pervasive pattern characterising macrobial communities^27^, and lends support to the notion of universal or unifying laws of assembly that apply as equally to microbes as they do to communities of plants and animals^23^. Assuming that the remaining (undiscovered) virodiversity was not detected because of rarity (that is, exists within the long tail of the rank abundance curve) and that rare viruses contribute little to the community^27^, we suggest that sufficient diversity has been detected with which to explore the structure of this naturally occurring community.

### Evidence of determinism driving viral community structure

**Figure 3:**
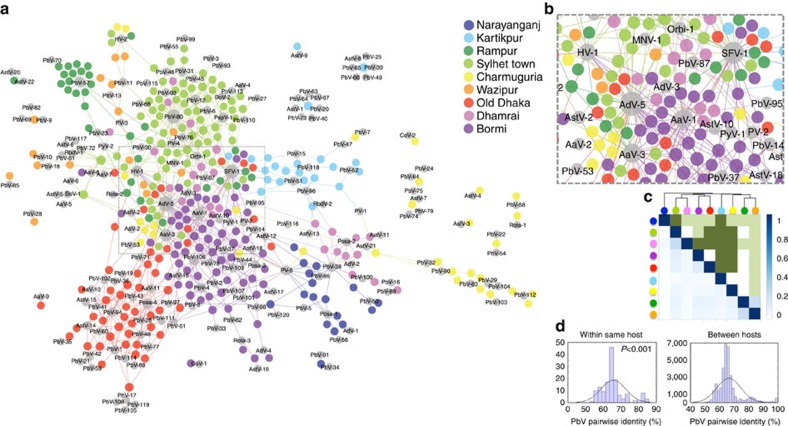
Two-mode affiliation network demonstrating the link between viruses and their hosts. Viruses shaded grey. Size of node indicates abundance. Coloured nodes indicate individual macaques, coloured by site. (**a**) The overall network shows a non-random pattern of site-associated diversity. (**b**) Insert showing an area of apparently stochastic distributions. (**c**) Lower corner (blue shading) shows a pairwise assessment of beta (β) diversity between sites. The Jaccard (incidence based) index was used and demonstrates that little similarity exists between sites. Additional metrics shown in Supplementary Fig. 1. Top corner (green shading) presents results of the null model, and shows that the observed distributions are different from chance (dark green=significant *P* value). (**d**) Distribution of pairwise genetic identities for PbVs found in the same host, and those found in different hosts. Results presented are for G1 PbVs, but results consistent for G2. *P* value (Wilcoxon rank-sum test) indicates that the ‘within same host' values (max=85.5%) are different from chance.

A two-mode affiliation network^28^ was used to illustrate the connectivity between viruses and their hosts. This revealed a dominant (though not exclusive) pattern of site-specific diversity consistent with determinism (Fig. 3a). To ensure that the patterns observed here were not simply the result of chance, null models^19^ were incorporated into an assessment of β-diversity^29^ (difference in viral composition between sites) and used to confirm that individual macaques mostly shared viruses with other individuals from the same site, and only rarely with those located elsewhere (Fig. 3c and Supplementary Fig. 1). By applying phylogenetic measures of β-diversity (Beta Nearest Taxon Index^30^,^31^) to a subset of the community (applied to the 120 PbVs) we were also able to infer that these non-random patterns may be emerging due to dispersal limitation (Supplementary Fig. 2).

To verify that dispersal (a largely stochastic process) was not responsible for the observed distributions, we correlated β-diversity (Jaccard index) with distance between sites to look further at the potential influence of dispersal limitation, and found no significant association (Mantel test: *P*=0.807; Principle Coordinates of Neighbour Matrices (PCNM)^31^,^32^,^33^: −0.352, *P*=0.134). We also tested for dispersal limitation by looking at whether PbV sequences from the same site were more related to each other than to viruses from other sites and whether this relatedness decreased with increasing site distance. When ‘same-site' (distance=0 km) was included in the analysis, the association was shown to be significant for both genotype 1 (G1) and genotype 2 (G2) PbVs (Spearman's rank correlation test; G1: *ρ*=−0.034; *P*<0.001; G2: *ρ*=−0.186; *P*<0.001). However when removed to test the strength of the effect, the significance of the correlation was lost (G1: *ρ*=−0.209; *P*=0.222; G2: *ρ*=−0.149; *P*=0.448). These results confirm that while there is substantial dispersal among macaques within a population, there is very limited dispersal among populations — regardless of geographic distance separating them. Evidence of multiple recombination events between viruses detected at different sites (Supplementary Table 2) and the natal migrations of male macaques seeking new groups^22^,^34^ both demonstrate connectivity between these populations, and suggest that these viruses are not (completely) limited in their ability to disperse. However, the frequency at which viruses become established in new populations via dispersal is seemingly low. Although we interpret these patterns of β-diversity as the result of deterministic processes based on our definition (that is, non-random), we also acknowledge that very low, or very high, rates of dispersal can lead to non-random patterns.

Determinism was also observed on more local scales, within sites and individuals. Using the PbV data (again, because of its presence at all sites) we looked to see whether there was a limit to how genetically similar two co-occurring viruses could be. The maximum observed identity between any two PbV sequences found in the same individual was 85.8% for G1 viruses, and 88.7% for G2 viruses (Fig. 3d). In contrast, the maximum identity for any two non-identical sequences found in different individuals at the same site was 99.8% (for both G1 and G2). This pattern was shown to be significantly different from chance (Wilcoxon rank-sum test; *P*<0.001) based on 1,000 random selections of γ-diversity (restricting α-diversity to the richness observed), and was consistent when stratified by site. It strongly suggests deterministic mechanisms do exist to limit the co-occurrence of closely related viruses in the same animal, and while the specific mechanisms are unknown we postulate they could well include virus:virus interactions such as competitive exclusion (analogous to the theory of limiting similarity^16^) or virus:host interactions like immune recognition. We qualify that this conclusion is dependent on the assumption that a correlation exists between phylogenetic relatedness and ecological similarity^30^ (for a competitive process) or host response (for immune recognition), and while we see no reason to doubt the validity of this assumption we acknowledge that little is currently known about picobirnavirus ecology and host interactions. We therefore suggest that additional data exploring whether ecological similarity increases with genetic similarity will now be required to confirm this relationship.

**Figure 4:**
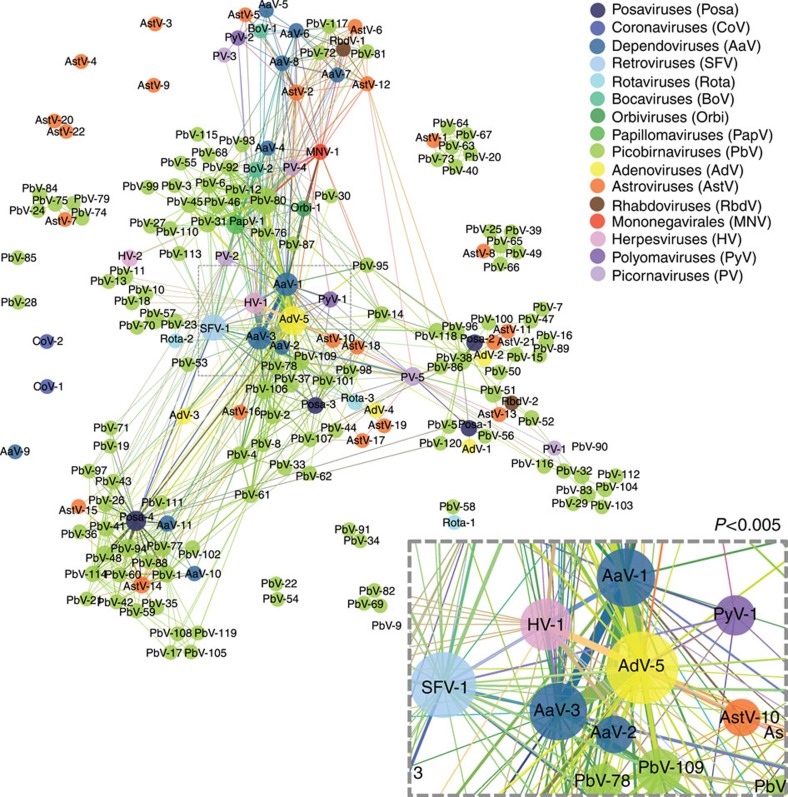
One-mode affiliation network demonstrating the frequency of viral co-occurrence in the same host. Viruses (coloured by family/genus) are linked if found in the same individual. Frequency of co-occurrence indicated by thickness of the edge connecting each node. Insert shows significant (*C*-score *P*<0.05) co-occurrence of AaVs with AdV and HV (significance determined using PAIRS).

The potential for virus:virus interactions was investigated further using a one-mode network that showed the connectivity of viruses based on the relative frequency of host sharing (Fig. 4). Several biological associations were apparent, including that of adenovirus MmAdV-5 with dependoviruses MmAaV-1, 2 and 3. Named dependoviruses (or adeno-associated viruses) because of their requirement for a ‘helper virus', these small DNA viruses are well known to use adenovirus to satisfy their replicative deficiencies^35^. The strength of this association was tested using PAIRS^24^,^36^, and the frequency of their co-occurrence shown to be significantly greater than expected by chance (*C*-score; *P*<0.001). The network also identified significant co-occurrence between MmAaV-1 and the herpesvirus MmHV-1 (*P*=0.002). Herpesviruses are also known to satisfy the helper requirements of dependoviruses^35^. Together these results demonstrate (and to some degree, validate) the usefulness of networks in understanding biological relationships in viral communities. In total, 35/184 viruses showed statistically supported (*P*=<0.05) positive co-occurrence with another virus, while 12/184 had negative associations. These results demonstrate that deterministic mechanisms exist to both promote and prevent the co-occurrence of viruses in the community.

### Stochastic distributions

Not all distributions could be attributed to determinism. MmAdV-5 was shown to significantly co-occur with various dependoviruses, but no discernible pattern could be identified and tested to explain its own distribution (that is, the presence of MmAdV-5 would explain the presence of MmAaV-1, 2 and 3, but perhaps not vice versa). The same is true for simian foamy virus (MmSFV) and the two HVs (MmHV-1 and 2), which like MmAdV-5 were detected at multiple sites without any apparently deterministic signature (Fig. 3b). We therefore attribute the distribution of these viruses to stochastic processes but acknowledge that scale or incomplete sampling might be obscuring determinism^18^.

## Discussion

Our results suggest that viral communities in the rhesus macaque are heavily influenced by deterministic factors, and therefore likely to be inherently structured. The effects of determinism were not absolute however, as stochastic processes also appeared to contribute to virodiversity. As such, we conclude that it should be possible to forecast changes to a significant portion of the viral community in a given location, but suggest there will also be some portion for which prediction will always be unlikely. We qualify that our study only demonstrates that changes in viral diversity should eventually be predictable, based on the assumption that non-random patterns in biological systems infer inherent predictability^16^,^18^,^19^,^23^,^37^, and based on the assumption that our data is representative of the entire community (that is, including those viruses that were not discovered, and assumed to be rare). It does not, and is not intended to, present a framework for how this prediction might be achieved. Instead, this study contributes to the theory that will support the future development of these probabilistic models, describing how the distribution of viruses is likely to change in response to different environmental or host factors. To achieve this, we advocate investigating the specific mechanisms associated with determinism (for example, the host:host; host:virus or virus:virus interactions) as well as the continued and systematic description of wildlife virodiversity through time and space. We also acknowledge that this finding will not lead directly to predictions of disease emergence, however we suggest it does provide the basis on which to test the hypothesis that drivers such as land-use change or climate (among others) promote disease emergence through their effect on the structure of the zoonotic pool.

## Methods

### Sample collection

Faecal samples (*n*=458) were collected non-invasively from free-ranging rhesus macaques (*Macaca mulatta*) over a 2-month period (February/March-2013) under ethical approval from the International Centre for Diarrhoeal Disease Research, Bangladesh (icddr, b; protocol: 2008-074) and UC Davis (protocol: 16048). Location of each site and a description of the macaque populations are provided in Supplementary Table 3. Sampling effort was consistent for all sites (3 days per site). All samples were collected immediately after defecation and stored in liquid nitrogen within 10 min of collection, until transfer to −80 °C for storage.

### Sample processing and viral discovery

Samples were viral particle enriched through (i) filtration to remove cellular debris and bacteria and (ii) nuclease treatment to remove unencapsulated RNA/DNA. For this, samples were thawed on ice and 500 μl of viral transport medium (Viral Transport Medium (VTM); BD Universal Viral Transport System) added, vortexed to homogenise, and centrifuged for 5 min at 8,000*g*. Supernatant was transferred to an Ultrafree-MC HV Centrifugal Filter 0.45 μM (Milipore Cat. No. UFC30HVNB) and centrifuged for 3 min at 12,000g. The flow (∼130–150 μl) was collected and 1 μl RNase A (Ribonuclease protection assay Grade, 1 mg ml^−1^, Life Technologies Cat. No. AM2272) added and incubated at room temperature for 15 min. If the flow volume was close to 200 μl then 2 μl of RNase A was used. Following RNase treatment, 1.5 μl of MgCl_2_ (1M), 4 μl of Turbo DNase (2 U ml^−1^, Ambion Cat. No. AM2238) and 1 μl of Benzonase. (Novagen, Cat. No. 70664-3), were added, mixed gently and incubated at room temperature for 45 min. Roche MagNa Pure lysis solution was added immediately to inactivate nucleases and lyse viral particles, and total nucleic acids extracted using the Roche MagNA Pure 96 platform according to the manufacturer's instructions.

Samples were processed for viral detection and discovery using both consensus PCR and next-generation sequencing. cPCR, allows the ‘universal' amplification of sequences from viruses within a given family or genus, and the subsequent discernment of viral strains within. Total nucleic acids was reverse transcribed into cDNA using SuperScript III (Invitrogen) according to the manufacturer's instructions, and a total of 41 assays representing 27 viral families or genera used for the detection of viral sequences. Two synthetic plasmids were constructed for use as ‘universal controls' to confirm successful execution of each assay and check for contamination (Supplementary Fig. 3). Detailed protocols for all cPCR assays used are provided in the Supplementary Methods and Supplementary Table 4. Bands of the expected size were excised from 1% agarose, cloned into Strataclone PCR cloning vector, and 24 white colonies sequenced to confirm detection and look for co-occurring viruses.

To guard against the potential of cPCR to miss viruses that are divergent or not among the targeted viral families, HTS was also applied to all samples. Although generally less sensitive than PCR, HTS allows for the capture of a very broad diversity of viruses because it amplifies all viral nucleic acids present. Samples were processed in pools of eight, and libraries prepared for both the Ion Torrent (PGM; 1 million reads per pool) and Illumina (High-Seq; 10 million reads per pool) platforms, according to each of the manufacturer's instructions. Sequence reads were aligned against host reference databases to remove host background using bowtie2 mapper, and host-subtracted reads primer trimmed and filtered based on quality, GC content and sequence complexity. The remaining reads were *de novo* assembled using Newbler (v2.6) for PGM data and MIRA (v4.0) for Illumina. Contigs and unique singletons were subjected to homology search using MegaBlast against the GenBank nucleotide database. Sequences that showed poor or no homology at the nucleotide level were blasted using BLASTx against the viral GenBank protein database. Viral sequences from the BLASTx analysis were subjected to a homology search against the GenBank protein database to correct for biased e-values. Sequences of plant viruses or insect viruses from viral families that have (to date) never been associated with infection of any vertebrate species were not considered in this study. All other viral sequences identified by HTS were subsequently confirmed by PCR and all samples re-screened individually to assess sequence distribution in all macaques (as we assume the lower sensitivity of HTS may produce false negatives). Where substantial diversity was observed (for example, PbVs), new cPCR assays were designed based on the HTS data, and used to re-screen all pooled samples individually to detect the full diversity present.

### Network, phylogenetic and statistical analyses

A presence/absence matrix was constructed to show the distribution of viruses across all samples, and used for network and statistical analyses as described in the main text. In summary, A bipartite (two-mode) affiliation network was generated for virus–macaque host matrix data, stratified by site name, and a unipartite (one-mode) virus:virus network was generated to display the connections between viruses. Network analyses and visualisation were conducted in the network analysis platform Gephi, using the force-directed algorithm ForceAtlas2 (ref. 28). Significance of pairwise associations was determined using PARS^24^,^36^. All other statistical analyses were performed using MATLAB (Mathworks, Natick USA) version R2013a. Discovery curves were generated in R package iNEXT. Phylogenetic analyses of sequence data were performed using MUSCLE^38^ for initial alignments, followed by manual refinement in Se-Al v2.0a11 (ref. 39). Maximum Likelihood trees were reconstructed using PAUP* (ref. 40) and best fitting models selected using jModeltest v2.1.5 (ref. 41). Trees were annotated using iTOL (v2.1)^42^. Measures of phylogenetic β-diversity were performed by first calculating the genetic similarity between every two different PbV sequences. This similarity matrix was then transformed into a distance matrix using the method proposed by Dray *et al*.^31^, which was then used to calculate the βMNTD (Beta Mean Nearest Taxon Distance)^30^. Results were compared with a null distribution of βMNTD, where PbV taxa were randomised across sites with a fixed relative abundance and recalculated 999 times. Beta Nearest Taxon Index values were then calculated as the number of s.d. that the observed βMNTD is from the mean of the null distribution.

## Additional information

**Accession codes**: The sequence data have been deposited in the GenBank nucleotide database under accession codes KT599642 to KT599859, KT334810 to KT335259, and KT599483 to KT599639.

**How to cite this article:** Anthony, S. J. *et al*. Non-random patterns in viral diversity. *Nat. Commun.* 6:8147 doi: 10.1038/ncomms9147 (2015).

## Supplementary Material

Supplementary Information Supplementary Figures 1-3, Supplementary Tables 1-4, Supplementary Methods and Supplementary References
