# Supplementary material for: Non-random patterns in viral diversity
Source: Nat Commun. 2015 Sep 22;6:8147. doi: 10.1038/ncomms9147 (PMC4595600; doi:10.1038/ncomms9147)
Supplement: Supplementary Information — Supplementary Figures 1-3, Supplementary Tables 1-4, Supplementary Methods and Supplementary References [file ncomms9147-s1.pdf]

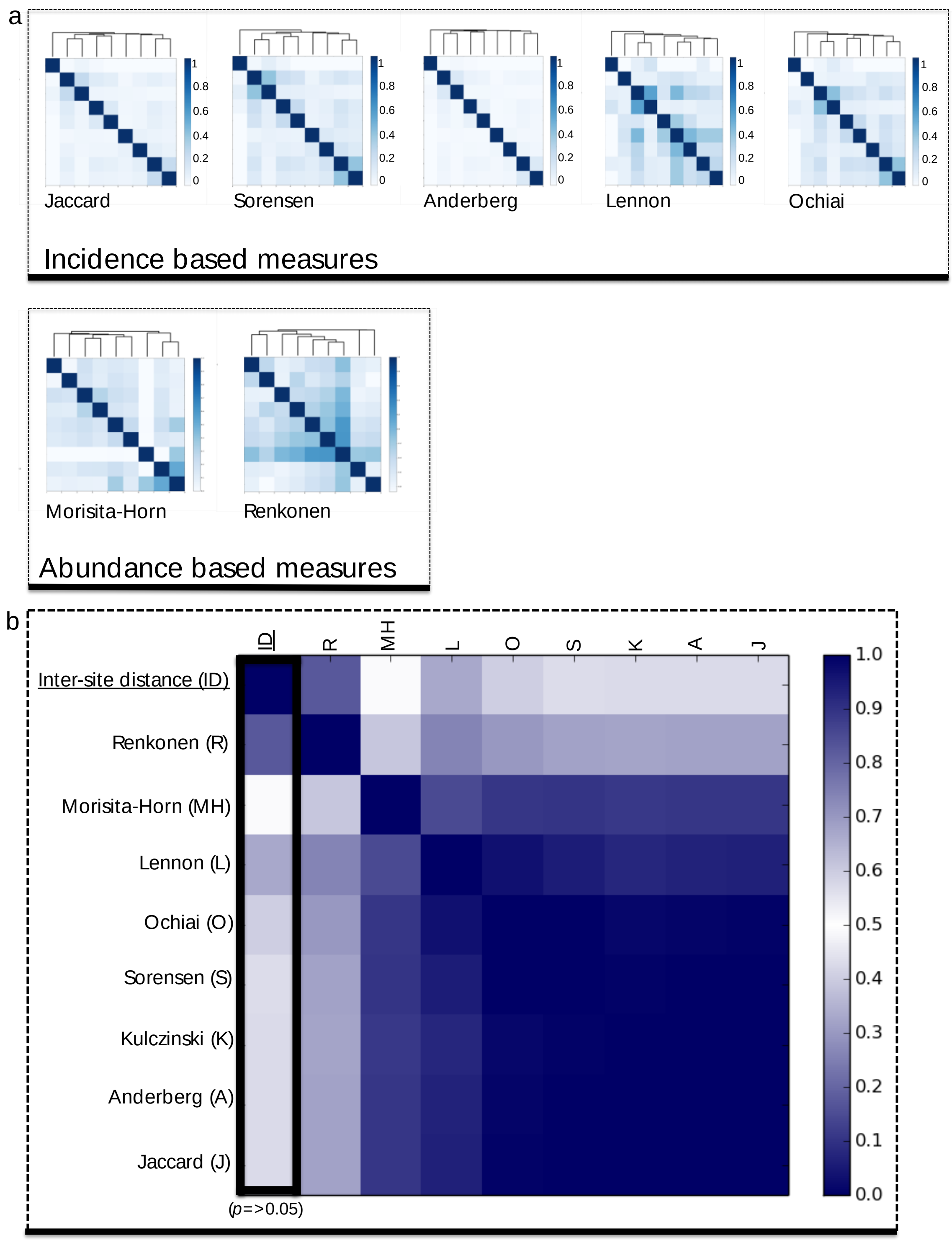

Supplementary Figure 1:

Compositional similarity between sites assessed using several metrics of beta (β) diversity (both incidence and abundance based methods). Mantel test is used to

correlate results for each. General agreement is observed and Jaccard index is selected for incorporation in the null model (see Fig 3).

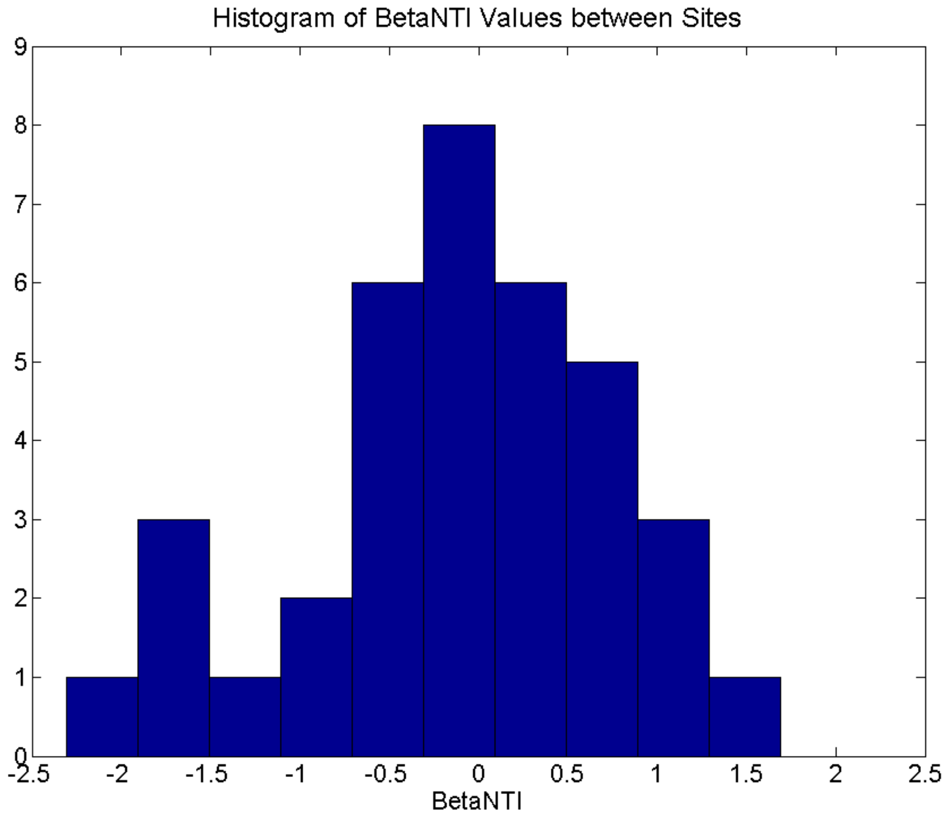

Supplementary Figure 2:

Histogram showing the distribution of β-Nearest Taxon Index (β-NTI) values. If the β-NTI distribution is significantly shifted towards -2 or +2, it would indicate an important role for deterministic selective forces. Here we show that the distribution is not significantly shifted from zero, which in combination with the Jaccard-based null modelling (see Fig 3) suggests that non-random patterns may be emerging due to dispersal limitation.

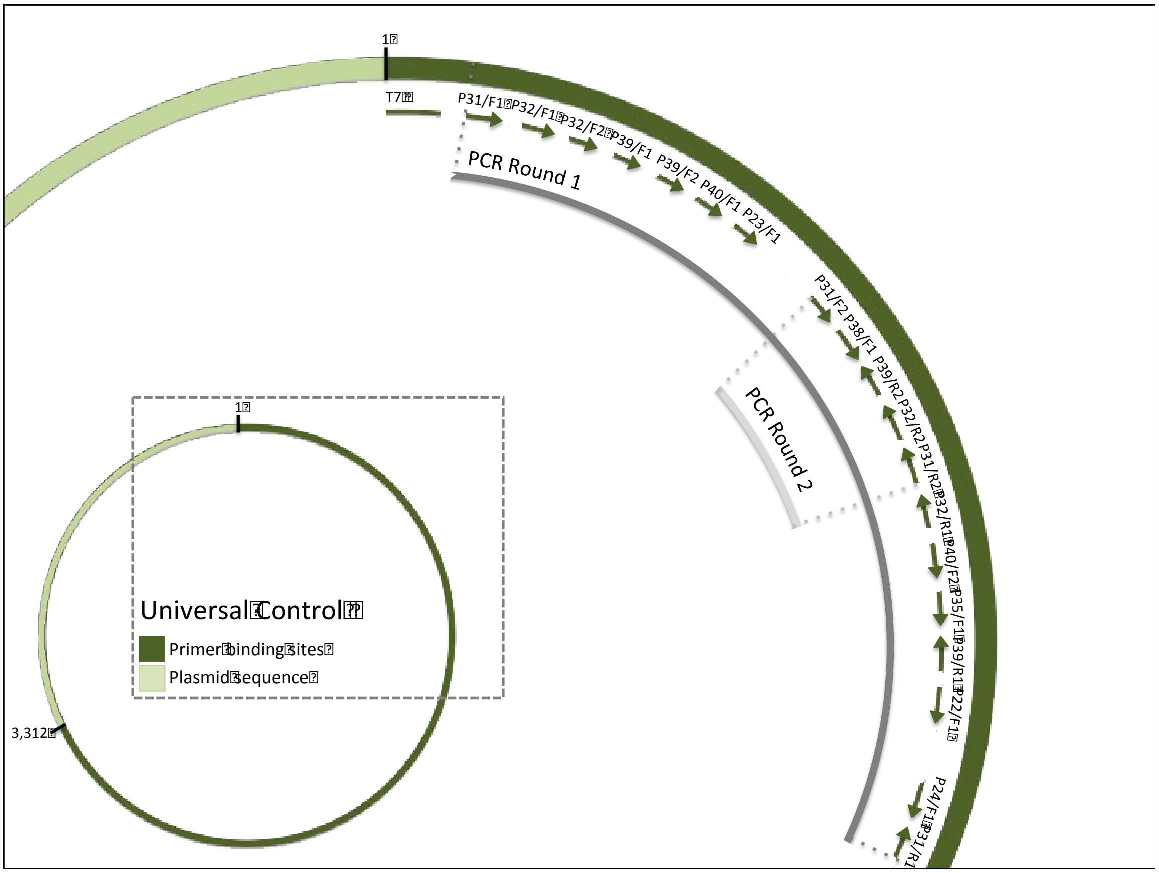

Supplementary Figure 3:

Schematic of Universal Control 2. Two constructs (UC1 and UC2) were generated to provide positive control material for the consensus PCR screening. These constructs are made up of sequential (non-overlapping) primer-binding sites for all assays, interspersed with short stretches of synthetic sequence. UC2 is shown here as an example to illustrate the structure of these controls. UC1 and UC2 allow the amplification of PCR products of the expected size in order to confirm successful execution of the assay. They also allow easy recognition of sample contamination, given that amplified products contain a series of primer-binding sites rather than a real viral sequence. Protocol P-031 (herpesvirus) is shown as an example. Constructs include a T7-promoter sequence at the beginning for transcription into RNA templates where desired.

				Method of Detection			
**Virus**	**Known/Novel**	**Interpretation**	**Abundance**	**PCR**	**Deep-Sequencing (PCR confirmed)**	**Sequence length (# bp)**	**Accession Numbers**
MmAdV-1	Novel	PREDICT_AdV-69	1	1	0	275	KT599483
MmAdV-2	Novel	PREDICT_AdV-72	3	1	0	275	KT599484-KT599486
MmAdV-3	Novel	Simian Adenovirus	6	1	0	275	KT599487-KT599492
MmAdV-4	Known	Human Adenovirus F	3	1	0	278	KT599493-KT599495
MmAdV-5	Known	Human Adenovirus G	54	1	1	278	KT599496-KT599560
MmBoV-1	Known	Human Bocavirus 3	1	1	0	260	KT599830
MmBoV-2	Novel	Feline Bocavirus	6	0	1	292	KT599602-KT599607
MmCoV-1	Novel	PREDICT_CoV-49	1	1	0	293	KT599733
MmCoV-2	Novel	PREDICT_CoV-50	1	1	0	293	KT599734
MmPicornaV-1	Known	Human Enterovirus B	2	1	0	315	KT599708-KT599709
MmPicornaV-2	Novel	PREDICT_PicornaV-1	5	0	1	544	KT599831-KT599835
MmPicornaV-3	Novel	PREDICT_PicornaV-2	2	0	1	544	KT599836-KT599837
MmPicornaV-4	Novel	PREDICT_PicornaV-3	7	0	1	328-380	KT599838-KT599844
MmPicornaV-5	Novel	PREDICT_PicornaV-4	15	0	1	376-410	KT599846-KT599859
MmSFV-1	Known	Macaque Simian Foamy Virus	44	1	0	285-580	KT599672-KT599705 KT599710-KT599727
MmHV-1	Known	Macacine Herpesvirus 4	23	1	0	177-378	KT599642-KT599671
MmHV-2	Known	Macacine Herpesvirus 3	8	0	1	202	KT599608-KT599615
MmPyV-1	Known	BK Polyomavirus	6	1	1	218-360	KT599616-KT599621 KT599829
MmPyV-2	Novel	PREDICT_PyV-31	3	1	0	221	KT599738-KT599740
MmAstV-1	Novel	PREDICT_MAstV-137	1	1	0	306	KT599561
MmAstV-2	Novel	PREDICT_MAstV-122	6	1	0	288	KT599562-KT599567

MmAstV-3	Novel	PREDICT_MAstV-111	1	1	0	288	KT599568
MmAstV-4	Novel	PREDICT_MAstV-111	1	1	0	288	KT599569
MmAstV-5	Novel	PREDICT_MAstV-112	1	1	0	288	KT599570
MmAstV-6	Novel	PREDICT_MAstV-113	1	1	0	288	KT599571
MmAstV-7	Novel	PREDICT_MAstV-117	1	1	0	294	KT599572
MmAstV-8	Novel	PREDICT_MAstV-118	1	1	0	300	KT599573
MmAstV-9	Known	Mamastrovirus 1	1	1	0	288	KT599574
MmAstV-10	Known	Mamastrovirus 5	2	1	0	288	KT599575-KT599576
MmAstV-11	Known	Mamastrovirus 5	1	1	0	288	KT599577
MmAstV-12	Known	Mamastrovirus 6	2	1	0	282	KT599578-KT599579
MmAstV-13	Known	Mamastrovirus 6	2	1	0	282	KT599580-KT599581
MmAstV-14	Novel	PREDICT MAstV-125	9	1	0	312	KT599582-KT599590
MmAstV-15	Novel	PREDICT MAstV-125	1	1	0	312	KT599591
MmAstV-16	Novel	PREDICT MAstV-126	3	1	0	312	KT599592-KT599594
MmAstV-17	Novel	PREDICT MAstV-126	1	1	0	312	KT599595
MmAstV-18	Novel	PREDICT MAstV-127	1	1	0	312	KT599596
MmAstV-19	Novel	PREDICT MAstV-128	1	1	0	309	KT599597
MmAstV-20	Known	Avastrovirus 2	1	1	0	312	KT599598
MmAstV-21	Known	Avastrovirus 2	2	1	0	309	KT599599-KT599600
MmAstV-22	Known	Avastrovirus 2	1	1	0	309	KT599601
MmPbV-1	Novel	PREDICT_PbV-1	11	0	1	544	KT334810-KT334820
MmPbV-2	Novel	PREDICT_PbV-2	6	0	1	544	KT334821-KT334826
MmPbV-3	Novel	PREDICT_PbV-3	1	0	1	544	KT334827
MmPbV-4	Novel	Closely related to a known human PbV	14	0	1	544	KT334828-KT334841
MmPbV-5	Novel	Closely related to a known human PbV	2	0	1	544	KT334842-KT334843
MmPbV-6	Novel	PREDICT_PbV-6	9	0	1	544	KT334844-KT334852
MmPbV-7	Novel	PREDICT_PbV-7	1	0	1	544	KT334853

MmPbV-8	Novel	PREDICT_PbV-8	2	0	1	544	KT334854-KT334855
MmPbV-9	Novel	PREDICT_PbV-9	1	0	1	544	KT334856
MmPbV-10	Novel	PREDICT_PbV-10	3	0	1	544	KT334857-KT334859
MmPbV-11	Novel	PREDICT_PbV-11	6	0	1	544	KT334860-KT334865
MmPbV-12	Novel	PREDICT_PbV-12	17	0	1	544	KT334866-KT334882
MmPbV-13	Novel	PREDICT_PbV-13	2	0	1	544	KT334883-KT334884
MmPbV-14	Novel	PREDICT_PbV-14	7	0	1	541	KT334885-KT334891
MmPbV-15	Novel	PREDICT_PbV-15	3	0	1	544	KT334892-KT334894
MmPbV-16	Novel	PREDICT_PbV-16	2	0	1	550	KT334895-KT334896
MmPbV-17	Novel	PREDICT_PbV-17	1	0	1	544	KT334897
MmPbV-18	Novel	PREDICT_PbV-18	2	0	1	541	KT334898-KT334899
MmPbV-19	Novel	PREDICT_PbV-19	2	0	1	544	KT334900-KT334901
MmPbV-20	Novel	PREDICT_PbV-20	1	0	1	544	KT334902
MmPbV-21	Novel	Closely related to a known human PbV	1	0	1	544	KT334903
MmPbV-22	Novel	PREDICT_PbV-22	1	0	1	544	KT334904
MmPbV-23	Novel	PREDICT_PbV-23	4	0	1	544	KT334905-KT334908
MmPbV-24	Novel	PREDICT_PbV-24	1	0	1	532	KT334909
MmPbV-25	Novel	PREDICT_PbV-25	1	0	1	541	KT334910
MmPbV-26	Novel	PREDICT_PbV-26	4	0	1	544	KT334911-KT334914
MmPbV-27	Novel	PREDICT_PbV-27	1	0	1	544	KT334915
MmPbV-28	Novel	PREDICT_PbV-28	1	0	1	538	KT334916
MmPbV-29	Novel	PREDICT_PbV-29	2	0	1	544	KT334917-KT334918
MmPbV-30	Novel	PREDICT_PbV-30	1	0	1	544	KT334919
MmPbV-31	Novel	PREDICT_PbV-31	13	0	1	544	KT334920-KT334932
MmPbV-32	Novel	PREDICT_PbV-32	3	0	1	544	KT334933-KT334935
MmPbV-33	Novel	PREDICT_PbV-33	1	0	1	544	KT334936
MmPbV-34	Novel	PREDICT_PbV-34	1	0	1	544	KT334937

MmPbV-35	Novel	PREDICT_PbV-35	1	0	1	544	KT334938
MmPbV-36	Novel	PREDICT_PbV-36	1	0	1	544	KT334939
MmPbV-37	Novel	PREDICT_PbV-37	5	0	1	544	KT334940-KT334944
MmPbV-38	Novel	PREDICT_PbV-38	16	0	1	544	KT334945-KT334960
MmPbV-39	Novel	PREDICT_PbV-39	1	0	1	544	KT334961
MmPbV-40	Novel	PREDICT_PbV-40	1	0	1	544	KT334962
MmPbV-41	Novel	PREDICT_PbV-41	4	0	1	544	KT334963-KT334966
MmPbV-42	Novel	PREDICT_PbV-42	2	0	1	544	KT334967-KT334968
MmPbV-43	Novel	PREDICT_PbV-43	4	0	1	544	KT334969-KT334972
MmPbV-44	Novel	PREDICT_PbV-44	1	0	1	544	KT334973
MmPbV-45	Novel	PREDICT_PbV-45	5	0	1	474-544	KT334974-KT334978
MmPbV-46	Novel	PREDICT_PbV-46	5	0	1	544	KT334979-KT334983
MmPbV-47	Novel	PREDICT_PbV-47	2	0	1	544	KT334984-KT334985
MmPbV-48	Novel	PREDICT_PbV-48	7	0	1	542-544	KT334986-KT334992
MmPbV-49	Novel	PREDICT_PbV-49	1	0	1	546	KT334993
MmPbV-50	Novel	PREDICT_PbV-50	3	0	1	544	KT334994-KT334996
MmPbV-51	Novel	PREDICT_PbV-51	9	0	1	544	KT334997-KT335005
MmPbV-52	Novel	PREDICT_PbV-52	4	0	1	544	KT335006-KT335009
MmPbV-53	Novel	Closely related to a known human PbV	1	0	1	544	KT335010
MmPbV-54	Novel	PREDICT_PbV-54	1	0	1	544	KT335011
MmPbV-55	Novel	PREDICT_PbV-55	1	0	1	544	KT335012
MmPbV-57	Novel	PREDICT_PbV-57	18	0	1	544	KT335016-KT335030
MmPbV-58	Novel	PREDICT_PbV-58	2	0	1	544	KT335031-KT335032
MmPbV-59	Novel	PREDICT_PbV-59	2	0	1	544	KT335033-KT335034
MmPbV-60	Novel	PREDICT_PbV-60	3	0	1	544	KT335035-KT335037
MmPbV-61	Novel	PREDICT_PbV-61	7	0	1	544	KT335038-KT335044
MmPbV-62	Novel	PREDICT_PbV-62	1	0	1	544	KT335045

MmPbV-63	Novel	PREDICT_PbV-63	1	0	1	544	KT335046
MmPbV-64	Novel	PREDICT_PbV-64	1	0	1	544	KT335047
MmPbV-65	Novel	PREDICT_PbV-65	1	0	1	545	KT335048
MmPbV-66	Novel	PREDICT_PbV-66	1	0	1	545	KT335049
MmPbV-67	Novel	PREDICT_PbV-67	1	0	1	544	KT335050
MmPbV-68	Novel	PREDICT_PbV-68	2	0	1	544	KT335051-KT335052
MmPbV-69	Novel	Closely related to a known human PbV	1	0	1	386	KT335053
MmPbV-70	Novel	PREDICT_PbV-70	1	0	1	544	KT335054
MmPbV-71	Novel	PREDICT_PbV-71	1	0	1	571	KT335055
MmPbV-72	Novel	PREDICT_PbV-72	1	0	1	559	KT335056
MmPbV-73	Novel	PREDICT_PbV-73	1	0	1	535	KT335057
MmPbV-74	Novel	PREDICT_PbV-74	1	0	1	544	KT335058
MmPbV-75	Novel	PREDICT_PbV-75	2	0	1	532	KT335059-KT335060
MmPbV-76	Novel	PREDICT_PbV-76	3	0	1	544	KT335061-KT335063
MmPbV-77	Novel	PREDICT_PbV-77	7	0	1	544	KT335064-KT335070
MmPbV-78	Novel	PREDICT_PbV-78	5	0	1	544	KT335071-KT335075
MmPbV-79	Novel	PREDICT_PbV-79	1	0	1	544	KT335076
MmPbV-80	Novel	PREDICT_PbV-80	46	0	1	538-562	KT335077-KT335121
MmPbV-81	Novel	PREDICT_PbV-81	1	0	1	553	KT335122
MmPbV-82	Novel	PREDICT_PbV-82	1	0	1	544	KT335123
MmPbV-83	Novel	PREDICT_PbV-83	6	0	1	584-592	KT335124-KT335129
MmPbV-84	Novel	PREDICT_PbV-84	1	0	1	547	KT335130
MmPbV-85	Novel	PREDICT_PbV-85	1	0	1	547	KT335131
MmPbV-86	Novel	PREDICT_PbV-86	4	0	1	547	KT335132-KT335135
MmPbV-87	Novel	PREDICT_PbV-87	2	0	1	547	KT335136-KT335137
MmPbV-88	Novel	PREDICT_PbV-88	3	0	1	547	KT335138-KT335140
MmPbV-89	Novel	PREDICT_PbV-89	2	0	1	592	KT335141-KT335142

MmPbV-90	Novel	PREDICT_PbV-90	4	0	1	541	KT335143-KT335146
MmPbV-91	Novel	PREDICT_PbV-91	1	0	1	541	KT335147
MmPbV-92	Novel	PREDICT_PbV-92	5	0	1	541	KT335148-KT335152
MmPbV-93	Novel	PREDICT_PbV-93	1	0	1	541	KT335153
MmPbV-94	Novel	PREDICT_PbV-94	15	0	1	541	KT335154-KT335168
MmPbV-95	Novel	PREDICT_PbV-95	3	0	1	619	KT335169-KT335171
MmPbV-96	Novel	PREDICT_PbV-96	7	0	1	487-619	KT335172-KT335178
MmPbV-97	Novel	PREDICT_PbV-97	1	0	1	646	KT335179
MmPbV-98	Novel	PREDICT_PbV-98	3	0	1	616	KT335180-KT335182
MmPbV-99	Novel	PREDICT_PbV-99	1	0	1	599	KT335183
MmPbV-100	Novel	PREDICT_PbV-100	1	0	1	613	KT335184
MmPbV-101	Novel	PREDICT_PbV-101	8	0	1	599-613	KT335185-KT335192
MmPbV-102	Novel	PREDICT_PbV-102	3	0	1	613	KT335193-KT335195
MmPbV-103	Novel	PREDICT_PbV-103	1	0	1	559	KT335196
MmPbV-104	Novel	PREDICT_PbV-104	3	0	1	559	KT335197-KT335199
MmPbV-105	Novel	PREDICT_PbV-105	1	0	1	565	KT335200
MmPbV-106	Novel	PREDICT_PbV-106	16	0	1	559	KT335201-KT335216
MmPbV-107	Novel	PREDICT_PbV-107	4	0	1	452-559	KT335217-KT335220
MmPbV-108	Novel	PREDICT_PbV-108	1	0	1	559	KT335221
MmPbV-109	Novel	PREDICT_PbV-109	13	0	1	507-559	KT335222-KT335234
MmPbV-110	Novel	PREDICT_PbV-110	2	0	1	565	KT335235-KT335236
MmPbV-111	Novel	PREDICT_PbV-111	1	0	1	631	KT335237
MmPbV-112	Novel	PREDICT_PbV-112	3	0	1	513-571	KT335238-KT335240
MmPbV-113	Novel	PREDICT_PbV-113	2	0	1	571	KT335241-KT335242
MmPbV-114	Novel	PREDICT_PbV-114	6	0	1	571	KT335243-KT335248
MmPbV-115	Novel	PREDICT_PbV-115	1	0	1	598	KT335249
MmPbV-116	Novel	PREDICT_PbV-116	2	0	1	517-523	KT335250-KT335251

MmPbV-117	Novel	PREDICT_PbV-117	1	0	1	523	KT335252
MmPbV-118	Novel	PREDICT_PbV-118	4	0	1	520	KT335253-KT335256
MmPbV-119	Novel	PREDICT_PbV-119	1	0	1	547	KT335257
MmPbV-120	Novel	PREDICT_PbV-120	1	0	1	633	KT335258
MmOrbiV-1	Novel	PREDICT_Orbi-6	5	0	1	439-501	KT599728-KT599732
MmRotaV-1	Known	Rotavirus A	1	0	1	357	KT599706
MmRotaV-2	Known	Rotavirus A	1	0	1	357	KT599707
MmRotaV-3	Known	Rotavirus A	3	0	1	396-402	KT599735-KT599737
MmAaV-1	Known	Adeno-Associated Virus Clade A	33	0	1	364	KT599741-KT599773
MmAaV-2	Known	Adeno-Associated Virus Clade D	2	0	1	364	KT599774-KT599775
MmAaV-3	Known	Adeno-Associated Virus rh.8	32	0	1	364	KT599776-KT599807
MmAaV-4	Known	Adeno-Associated Virus Clade C	2	0	1	364	KT599808-KT599809
MmAaV-5	Novel	PREDICT_AaV-5	1	0	1	370	KT599810
MmAaV-6	Novel	PREDICT_AaV-6	2	0	1	370	KT599811-KT599812
MmAaV-7	Novel	PREDICT_AaV-7	2	0	1	370	KT599813-KT599814
MmAaV-8	Novel	PREDICT_AaV-8	4	0	1	370	KT599815-KT599818
MmAaV-9	Novel	PREDICT_AaV-9	1	0	1	364	KT599819
MmAaV-10	Novel	PREDICT_AaV-10	3	0	1	379	KT599820-KT599822
MmAaV-11	Novel	PREDICT_AaV-11	6	0	1	364	KT599823-KT599828
MmPapV-1	Novel	PREDICT_PapV-1	18	0	1	423	KT599622-KT599639

Supplemental Table 1:

Description of all viruses discovered in macaques: A total of 184 viruses were identified using a combination of consensus PCR (cPCR) and high-throughput sequencing (HTS). All viruses identified by HTS (contigs or singletons) were subsequently confirmed by PCR or cPCR and the length of this confirmed sequence is indicated. For some viruses, putative partial or near complete genomes were assembled from the HTS data (*). Note: a large diversity of picobirnaviruses (PbVs) was discovered in these macaques. Initially, approximately 50 different PbV sequences were detected in the HTS data. An alignment of these sequences was then used to design a novel cPCR assay (see P-030 in Supplemental Table 4), which was used to re-screen all samples individually by cPCR for the detection of all 120 viruses presented here. We therefore attribute the discovery of these viruses to HTS because no PbV assay used in the initial cPCR screen and none of them would have been discovered without the initial HTS data – but also note that the full diversity of PbVs discovered is actually the result of a combined HTS/cPCR strategy.

Recombinant	Recombination Event<br>Major Parent	Minor Parent	% Identity	Algorithm<br>RDP	GENECONV	BootScan	MaxChi	Chimaera	SiScan	3Seq
G1_MmPbV-65 (PRB_1201)	G1_MmPbV-63 (PRB_1199)	G1_MmPbV-1 (PRB_884)	77	**	**				***	
G1_MmPbV-1 (PRB_862)	G1_MmPbV-77 (PRB_875)	G1_MmPbV-1 (PRB_854)	71	***	***	***	***	***	***	***
G1_MmPbV-66 (PRB_1201)	G1_MmPbV-63 (PRB_1199)	G1_MmPbV-1 (PRB_884)	77	**	**				***	
G1_MmPbV-67 (PRB_1199)	G1_MmPV-63 (PRB_1199)	G1_MmPbV-1 (PRB_884)	77	**	**				***	
G1_MmPbV-28 (PRB_1125)	G1_MmPbV-9 (PRB_1128)	G1_MmPbV-53 (PRB_1177)	77	**	***	***	***		*	
G2_MmPbV-112 (PRB_1144)	G2_MmPbV-114 (PRB_891)	G2_MmPbV-112 (PRB_1184)	75	***	***	***	***	***	***	***
G2_MmPbV-112 (PRB_1147)	G2_MmPbV-114 (PRB_891)	G2_MmPbV-112 (PRB_1184)	44	***	***	***	***	***	***	***

*P<0.05, **P<0.01, ***P<0.001 (all P values are Bonferroni corrected)

Supplementary Table 2:

Evidence of intragenic recombination detected in the picobirnaviruses (PbVs). Sequences assessed using several algorithms (n=7), and putative recombination events accepted if significant for ≥3. Only significant recombinants presented. Coloured nodes indicate site (see figures 3 or 4 for site names).

Site	GPS	Est Pop Size	No. Groups	Mean Group Size	# Sampled
Old Dhaka	23.42192;90.25477	159	4	39.8 ± 13.8	65
Narayanganj	23.36903;90.30716	55	2	27.5 ± 3.5	20
Dhamrai	23.55056;90.12637	103	2	51.5 ± 4.9	34
Bormi	24.14669;90.31301	102	2	51.0 ± 5.6	77
Rampur	24.23586;90.71603	81	3	27.0 ± 3.0	66
Sylhet Town	24.90863;91.87811	260	5	52.0 ± 24.6	88
Charmuguria	23.17903;90.16905	210	3	70.0 ± 20.0	54
Wazipur	22.82619;90.24949	98	3	37.2 ± 3.8	22
Kartikpur	23.24258;90.47502	36	1	36.0 ± 0.0	32

Supplementary Table 3:

A description of the sites included in this study, including locations and macaque population sizes.

Assay	Family/Genus	Target	Reference	Amplicon (bp)	PCR Enzyme	Control
P-001	Astrovirus	RdRp	Atkins	~342	QIAGEN Fast Cycling Chemistry	UC2
P-002	Astrovirus	RdRp	Chu	421	QIAGEN Fast Cycling Chemistry	UC2
P-003	Arenavirus	S	Lozano	640	QIAGEN Fast Cycling Chemistry	UC1
P-004	Arenavirus	S	Lozano	460	QIAGEN Fast Cycling Chemistry	UC1
P-005	Coronavirus	RdRp	Quan	~328	QIAGEN Fast Cycling Chemistry	UC1
P-006	Coronavirus	RdRp	Watanabe	434	QIAGEN Fast Cycling Chemistry	UC1
P-007	Filovirus	L	Zhai	600	QIAGEN Fast Cycling Chemistry	UC1
P-008	Filovirus	L	Zhai	~600	QIAGEN Fast Cycling Chemistry	UC1
P-009	Flavivirus	NS5	Moureau	~270	QIAGEN Fast Cycling Chemistry	UC1
P-010	Flavivirus	NS5	Sanchez-Seco	141	QIAGEN Fast Cycling Chemistry	UC2
P-011	Picornavirus	5' UTR	Woo	~112	AmpliTaq Gold 360	Picorna cDNA
P-012	Enterovirus	VP3/VP1	Nix	350-450	QIAGEN Fast Cycling Chemistry	UC2
P-013	Enterovirus	5' UTR	*This study*	~400	QIAGEN Fast Cycling Chemistry	UC2
P-014	Paramyxovirus	POL	Tong	~561	QIAGEN Fast Cycling Chemistry	UC1
P-015	Henipahvirus	L	Feldman	265	QIAGEN Fast Cycling Chemistry	UC2
P-016	Rhabdovirus	L	Bourhy	260	QIAGEN Fast Cycling Chemistry	UC2
P-017	Lyssavirus	NP	Vazquez-Moron	259	QIAGEN Fast Cycling Chemistry	UC2

P-018	Seadornavirus	Segment 1	*This study*	747	QIAGEN Fast Cycling Chemistry	UC1
P-019	Alphavirus	NSP4	Sanchez-Seco	195	QIAGEN Fast Cycling Chemistry	UC1
P-020	Hantavirus	L	Klempa	~412	QIAGEN Fast Cycling Chemistry	Hanta cDNA
P-021	Phlebovirus	L	Sanchez-Seco	244	QIAGEN Fast Cycling Chemistry	UC2
P-022	Orthobunyavirus	S	Briese	~930	QIAGEN Fast Cycling Chemistry	UC2
P-023	Orthobunyavirus	M	Briese	~898	QIAGEN Fast Cycling Chemistry	UC2
P-024	Calicivirus	Capsid	Reid	768	QIAGEN Fast Cycling Chemistry	Calici cDNA
P-025	Influenza A	M	Anthony	~240	AmpliTaq Gold 360	UC2
P-026	Influenza A	PB1	*This study*	422	QIAGEN Fast Cycling Chemistry	Influenza cDNA
P-027	Simian Foamy virus	RdRp	Goldberg	632	QIAGEN Fast Cycling Chemistry	UC2
P-028	Simian Foamy virus	LTR	GOLDBERG	314	QIAGEN Fast Cycling Chemistry	UC2
P-029	Lentivirus	RdRp	Clewey	194	QIAGEN Fast Cycling Chemistry	UC2
P-030	Picobirnavirus	Segment 2	*This study*	~800	QIAGEN Fast Cycling Chemistry	Picobirna cDNA
P-031	Herpesvirus	Polymerase	Van de Vante	215-315	QIAGEN Fast Cycling Chemistry	UC2
P-032	Herpesvirus	Terminase	Chmielewicz	419	QIAGEN Fast Cycling Chemistry	UC2
P-033	Bocavirus	NS1	Kapoor	290	QIAGEN Fast Cycling Chemistry	UC1
P-034	Poxvirus	Polymerase	Bracht	~543	QIAGEN Fast Cycling Chemistry	UC2
P-035	Poxvirus	Topoisomerase	Bracht	~344	QIAGEN Fast Cycling Chemistry	UC2
P-036	Orthopoxvirus	RPO18	Nitsche	204	QIAGEN Fast Cycling Chemistry	UC2
P-037	Parapoxvirus	B2L	Inoshima	235	QIAGEN Fast Cycling Chemistry	UC2
P-038	Papillomaviurs	L1	Forslund	476	QIAGEN Fast Cycling Chemistry	UC2
P-039	Adenovirus	Polymerase	Wellehan	~320	AmpliTaq Gold 360	UC2
P-040	Polyomavirus	VP1	Johne	249-273	QIAGEN Fast Cycling Chemistry	UC2
P-041	Polyomavirus	VP3	Johne	~390-460	QIAGEN Fast Cycling Chemistry	PyV DNA

Supplementary Table 4:

A summary of the consensus PCR assays used in this study (n=41). Table intended to be used for quick ('at-a-glance') review. Detailed description of all assays is given in the Supplementary Methods.

Supplemental Methods: Consensus PCR (cPCR) Assays

Below is a detailed description of all cPCR assays used in this study, accompanied by anecdotal notes that may be useful to investigators wishing to use these methods for their own purposes.

RNA Virus Protocols:

P-001 Astroviruses

REFERENCE: **Atkins, A. et al (2009)** [1]
TARGET: RNA-Dependent RNA Polymerase (RdRp)
CONTROL: Universal Control 2 (UC2)
ENZYME: QIAGEN Fast Cycling PCR Kit (Cat. No. 203745)

	PRIMERS	PROTOCOL	AMPLICON
ROUND 1	Astr4380F-GAYTGGRCNCGNTWYGATGGNACIAT<br>Astr4811R-GGYTTNACCCACATNCCAAA	95°C 5 min, then 45 cycles of 96°C for 8 sec, 45°C for 8 sec and 68°C for 15 sec. Finish with 72°C for 3 min	~431 bp
ROUND 2	Astr4380F Same as for first round<br>Astr4722R-ARNCKRTCATCNCCATA	95°C 5 min, then 45 cycles of 96°C for 8 sec, 45°C for 8 sec and 68°C for 12 sec. Finish with 72°C for 3 min	~342 bp (variable)

NOTES: This assay works well to detect novel astroviruses, however investigators should be aware that it does cross-react fairly readily and often produces false-positive bands of the expected size. Sequencing will be required to rule out false positives.

P-002 Astroviruses

REFERENCE: **Chu, D.K.W. et al (2008)** [2]
TARGET: RdRp
CONTROL: UC2
ENZYME: QIAGEN Fast Cycling PCR Kit (Cat. No. 203745)

	PRIMERS	PROTOCOL	AMPLICON
ROUND 1	AstroFWD1;GARTTYGATTGGRCKCGKTAYGA<br>AstroFWD2-GARTTYGATTGGRCKAGGTAYGA<br>AstroRVS1-GGYTTKACCCACATNCCRAA	95°C 5 min, then 40 cycles of 96°C for 5 sec, 50°C for 8 sec and 68°C for 15 sec. Finish with 72°C for 2 min	~436 bp
ROUND 2	AstroFWD3-CGKTAYGATGGKACKATHCC<br>AstroFWD4-AGGTAYGATGGKACKATH CC<br>AstroRVS1 Same as for first round	Same protocol for both rounds	~421 bp

NOTES: None

P-003 Arenaviruses

REFERENCE: **Lozano, M. *et al* (1997)** [3]
TARGET: S-gene
CONTROL: Universal Control 1 (UC1)
ENZYME: QIAGEN Fast Cycling PCR Kit (Cat. No. 203745)

	PRIMERS	PROTOCOL	AMPLICON
ROUND 1	ARS16V-GGCATWGANCCAAACTGATT<br>ARS1-CGCACCGGGGATCCTAGGC	95°C 5 min, then 40 cycles of 96°C for 5 sec, 55°C for 8 sec and 68°C for 20 sec. Finish with 72°C for 3 min	640 bp

NOTES: Many sets of primers are presented by Lozano[3]*l*, which describes a method for full sequence characterization of arenaviruses. Two sets of primers have been selected from the paper (P-003 here, and P-004 below).

P-004 Arenaviruses

REFERENCE: **Lozano, M. *et al* (1997)** [3]
TARGET: S-gene
CONTROL: UC1
ENZYME: QIAGEN Fast Cycling PCR Kit (Cat. No. 203745)

	PRIMERS	PROTOCOL	AMPLICON
ROUND 1	ARS3V;CATGACKMTGAATTYTGTGACA<br>ARS7C-Mod;ATRTGYCKRTGWGTTGG	95°C 5 min, then 40 cycles of 96°C for 5 sec, 55°C for 8 sec and 68°C for 15 sec. Finish with 72°C for 2 min	460 bp

NOTE: Many sets of primers are presented by Lozano[3],, which describes a method for full sequence characterization of arenaviruses. Two sets of primers have been selected from the paper (P-003 above, and P-004 here). A slightly modified version of the 7C primer has been designed based on a more recent alignment of arenaviruses.

P-005 Coronaviruses

REFERENCE: **Quan, P.L. *et al* (2010)** [4]
TARGET: RdRp
CONTROL: UC1
ENZYME: QIAGEN Fast Cycling PCR Kit (Cat. No. 203745)

	PRIMERS	PROTOCOL	AMPLICON
ROUND 1	CoV-FWD1; CGTTGGIACWAAYBTVCCWYTICARBTRGG<br>CoVRVS1;GGTCATKATAGCRTCAVMASWWGCNACATG	95°C 5 min, then 14 cycles of 96°C for 5 sec, 65°C (-1°C per cycle) for 8 sec and 68°C for 18 sec, the 35 cycles of of 96°C for 5 sec, 50°C for 8 sec and 68°C for 18 sec. Finish with 72°C for 3 min	~520 bp
ROUND 2	CoV-FWD2;GGCWCCWCCHGGNGARCAATT<br>CoV-RVS2;GGWAWCCCCAYTGYTGWAYRTC	95°C 5 min, then 14 cycles of 96°C for 5 sec, 65°C (-1°C per cycle) for 8 sec and 68°C for 12 sec, the 35 cycles of of 96°C for 5 sec, 50°C for 8 sec and 68°C for 12 sec. Finish with 72°C for 3 min	~328 bp

NOTE: This assay has been used to identify a large diversity of novel coronaviruses in different species.

P-006 Coronaviruses

REFERENCE: **Modified from Watanabe, S. *et al* (2010)** [5]
TARGET: RdRp
CONTROL: UC1
ENZYME: QIAGEN Fast Cycling PCR Kit (Cat. No. 203745)

	PRIMERS	PROTOCOL	AMPLICON
ROUND 1	CoV-FWD3;GGTTGGGAYTAYCCHAARTGTGA	95°C 5 min, then 15 cycles 96°C for 5 sec, 65°C (-1°C per cycle) for 8 sec and 68°C for 15 sec, then 35 cycles of 96°C for 5 sec, 50°C for 8 sec and 68°C for 15 sec. Finish with 72°C for 3 min	440 bp
	CoV-RVS3;CCATCATCASWYRAATCATCATA		
ROUND 2	CoV-FWD4/Bat;GAYTAYCCHAARTGTGAYAGAGC	Same protocol for both rounds	434 bp
	CoV-FWD4/Other; GAYTAYCCHAARTGTGAUMGWGC		
	CoV-RVS3 Same reverse primer as round 1		

NOTES: Primer sequences have been modified to increase the ability of the assay to detect widely variant CoVs. A second, hemi-nested step has also been added to increase sensitivity. Nested PCR should be performed using both forward primers.

P-007 Filoviruses

REFERENCE: **Modified from Zhai, J. *et al* (2007)** [6]
TARGET: L
CONTROLS: UC1
ENZYME: QIAGEN Fast Cycling PCR Kit (Cat. No. 203745)

	PRIMERS	PROTOCOL	AMPLICON
ROUND 1	Filo-U12683-A;TATTCTCYCTACAAAAGCATTGGG	95°C for 5 min, followed by 12 cycles of 96°C for 5 sec, 65°C for 8 sec (-1°C per cycle) and 68°C for 20 sec. Then 35 cycles of 96°C for 5 sec, 52°C for 8 sec and 68°C for 20 sec. Finish with 72°C for 5 min	~600 bp
	Filo-L13294-A;GCTTCTGCGAGTGTTTGGACATT		
	Filo-U12683-B;TATTTTCCATTCAAAAACACTGGG		
	Filo-L13294-B:GCTTCACAAAGTGTTTGAACATT		
	Filo-U12683-C;TATTTTCAATCCAAAAGCACTGGG		
	Filo-L13294-C;GCTTCGCAGAGGGTTTGGACATT		
	Filo-U12683-D;TATTCTCTGTTCAAAAACATTGGG		
	Filo-L13294-D;GCCTCACATAAAGTTTGGACATT		

NOTE: Designed as a one-step RT-PCR. Using synthetic standards, the sensitivity was calculated to be 50-500 copies, so even with one round of PCR, it should be quite sensitive. The primers are well validated for known filoviruses, but their lack of degeneracy might prevent detection of diverse or novel filoviruses.

P-008 Filoviruses

REFERENCE: **Modified from Zhai, J. *et al* (2007)** [6]
TARGET: L
CONTROLS: UC1
ENZYME: QIAGEN Fast Cycling PCR Kit (Cat. No. 203745)

	MODIFIED PRIMERS	PROTOCOL	AMPLICON
ROUND 1	Filo-MOD-FWD;TITTYTCHVTICAAAAICAYTGGG<br>Filo-MOD-RVS;GCYTCISMIAIIGTTTGIACATT	95°C for 5 min, followed by 12 cycles of 96°C for 5 sec, 65°C for 8 sec (-1°C per cycle) and 68°C for 20 sec. Then 35 cycles of 96°C for 5 sec, 52°C for 8 sec and 68°C for 20 sec. Finish with 72°C for 5 min	~600 bp

NOTE: This assay includes a modified set of primers (from P-007) with introduced degeneracy to improve their use for pathogen discovery.

P-009 Flaviviruses

REFERENCE: **Moureau, G. *et al,* (2007)** [7]
TARGET: NS5
CONTROL: UC1
ENZYME: QIAGEN Fast Cycling PCR Kit (Cat. No. 203745)

	PRIMERS	PROTOCOL	AMPLICON
ROUND 1	Flavi-FWD;TGYRBTTAYAACATGATGGG<br>Flavi-RVS;GTGTCCCAICCNGCNGTRTC	95°C for 5 min, followed by 40 cycles of 96°C for 5 sec, 50°C for 8 sec and 68°C for 9 sec. Finish with 72°C for 2 min	~270 bp

NOTE: Originally developed as a real-time (SYBR green) one-step RT-PCR method for the universal detection and identification of flaviviruses. However works well in a conventional PCR platform. The assay can be easily inhibited by too much nucleic acid, giving false negative results. Be sure to use <500ng/ul cDNA at all times.

P-010 Flaviviruses

REFERENCE: **Sanchez-Seco, M. P. et al (2005)** [8]
TARGET: NS5
CONTROL: UC2
ENZYME: QIAGEN Fast Cycling PCR Kit (Cat. No. 203745)

	PRIMERS	PROTOCOL	AMPLICON
ROUND 1	Flavi1POS;GAYYTIGGITGYGGIIGIGGIRGITGG<br>Flavi1NEG;TCCCAICCIGCIRTRTCRTCIGC	95°C 5 min, then 40 cycles of 96°C for 5 sec, 47°C for 8 sec and 68°C for 40 sec. Finish with 72°C for 5 min	1,384 bp
ROUND 2	Flavi2POS;YGYRTIYAYAWCAYSATGGG<br>Flavi2NEG;CCARTGITCYKYRTTIAIRAAICC	95°C 5 min, then 40 cycles of 96°C for 5 sec, 47°C for 8 sec and 68°C for 6 sec. Finish with 72°C for 2 min	141 bp

NOTE: This assay has not been as extensively validated as P-009, especially on clinical samples. However, it does contain a nested primer pair. In theory this should improve sensitivity, however the large size of the first round product may reduce the overall efficiency of this PCR. The size of the second round amplicon is very small. While this is still sufficient to confirm a positive result, we encourage investigators to run the PCR reactions from both rounds (on gels) as far more information can be gathered from the larger (round 1) product, if successfully amplified.

P-011 Picornavirus

REFERENCE: **Woo, P.C.Y. et al (2012)** [9]
TARGET: 5' UTR
CONTROLS: Picornavirus cDNA
ENZYME: AmpliTaq Gold 360 Master Mix (Applied Biosystems Cat. No. 439881)

	PRIMERS	PROTOCOL	AMPLICON
ROUND 1	FWD;GGACCCGTGAATGCGGCTAA	95°C 10 min, then 40 cycles of 94°C for 1 min, 60°C for 1 min and 72°C for 1 min. Finish with 72°C for 10 min	~112 bp
	RVS;CACGGAACACCGAAAGTAGT		

NOTE: Amplicon very small so remember to run on 2% gel.

P-012 Enteroviruses

REFERENCE: **Nix, W.A. (2006)** [10]
TARGET: VP3/VP1
CONTROLS: UC2
ENZYME: QIAGEN Fast Cycling PCR Kit (Cat. No. 203745)

	PRIMERS	PROTOCOL	AMPLICON
ROUND 1	224/FWD/VP3;GCIATGYTIGGIACICAYRT	95°C 5 min, then 40 cycles of 96°C for 5 sec, 42°C for 8 sec and 68°C for 30 sec. Finish with 72°C for 4 min	992 bp
	222/RVS/VP1;CICCIGGIGGIAYRWACAT		
ROUND 2	AN89/FWD/VP1;CCAGCACTGACAGCAGYNGARAYNGG	95°C 5 min, then 40 cycles of 96°C for 5 sec, 60°C for 8 sec and 68°C for 15 sec. Finish with 72°C for 2 min	350-450 bp
	AN88/RVS/VP1;TACTGGACCACCTGGNGGNAYRWACAT		

NOTE: Most assays for enteroviruses are validated heavily against human strains. This assay was used to amplify a dolphin enterovirus in 2009[11] so clearly demonstrates broad cross-reactivity. Its use has also been demonstrated on clinical samples. Amplicon size variable between enteroviruses. Investigators should note that this assay may cross react with human sequences and care should be taken with the interpretation of blast results.

P-013 Enteroviruses

REFERENCE: **Unpublished. Designed at CII.**
TARGET: 5' UTR
CONTROLS: UC2
ENZYME: QIAGEN Fast Cycling PCR Kit (Cat. No. 203745)

	PRIMERS	PROTOCOL	AMPLICON
ROUND 1	UTRLong-F;GGTCAAGCACTTCTGTTTCCC	95°C 5 min, then 16 cycles of 96°C for 5 sec, 65°C for 8 sec (-1°C/cycle) and 68°C for 15 sec. Then perform 32 cycles of 96°C for 5 sec, 48°C for 8 sec and 68°C for 15 sec. Finish with 72°C for 3 min	~400 bp
	UTRL541-R;GAAACACGGWCACCCAAAGTASTCG		

NOTE: Amplicon size variable between different enteroviruses. This assay was designed principally to target rhinoviruses. It will also cross react with other enteroviruses too, but is probably not sufficiently broad to detect all members of the genus.

P-014 Paramyxoviruses

REFERENCE: **Tong, S. *et al*, (2008)** [12]
TARGET: RdRp
CONTROL: UC1
ENZYME: QIAGEN Fast Cycling PCR Kit (Cat. No. 203745)

	PRIMERS	PROTOCOL	AMPLICON
ROUND 1	PAR-F1;GAAGGITATTGTCAIAARNTNTGGAC	95°C 5 min, then 40 cycles of 96°C for 5 sec, 48°C for 8 sec and 68°C for 20 sec. Finish with 72°C for 4 min	~639 bp
	PAR-R;GCTGAAGTTACIGGITCICCDATRTTNC		
ROUND 2	PAR-F2;GTTGCTTCAATGGTTCARGGNGAYAA	Same for both rounds	~561 bp
	PAR-R Same Reverse primer as round 1		

NOTE: The paper provides good validation of the primers against various paramyxoviruses, but when applied to clinical samples this assay can cross-react with host sequences quite readily. It has been observed that this is a particular problem with bat samples. When a paramyxovirus *is* present in a sample, the assay does seem to work well, and (mostly) amplifies a single and specific product, with very little non-specific amplification. Investigators should therefore proceed cautiously with any sample that looks to have products that are close to the right size, but which also have a high background of non-specific amplification. It is probable that such samples are in fact negative.

P-015 Henipavirus

REFERENCE: **Feldman, K. S. *et al* (2009)** [13]
TARGET: L
CONTROLS: UC2
ENZYME: QIAGEN Fast Cycling PCR Kit (Cat. No. 203745)

	PRIMERS	PROTOCOL	AMPLICON
ROUND 1	LFWD1;TGAGYATGTATATGAAAGATAAAGC	95°C 5 min, then 40 cycles of 96°C for 5 sec, 42°C for 8 sec and 68°C for 12 sec. Finish with 72°C for 3 min	363 bp
	LREV;TCATCYTTAACCATCCCGTTCTC		
ROUND 2	LFWD2;ACCGARCCAAGAAGATTGGT	95°C 5 min, then 40 cycles of 96°C for 5 sec, 46°C for 8 sec and 68°C for 9 sec. Finish with 72°C for 2 min	265 bp
	LREV Same as round 1		

NOTE: This paper presents multiple assays, both real-time and conventional. Here we present a conventional assay that should be broadly reactive for all henipaviruses, and which is hemi-nested for increased sensitivity. While focused on henipaviruses, investigators should note that this assay is not specific for henipaviruses, and will also pick up other paramyxoviruses.

P-016 Rhabdoviruses

REFERENCE: **Modified from Bourhy et al, 2005[14].**
TARGET: L
CONTROL: UC2
ENZYME: QIAGEN Fast Cycling PCR Kit (Cat. No. 203745)

	PRIMERS	PROTOCOL	AMPLICON
ROUND 1	PVO3;CCADMCBTTTTGYCKYARRCCTTC (RVS)<br>PVO4;RAAGGYAGRTTTTTYKCDYTRATG (FWD)	95°C 5 min, then 14 cycles of 96°C for 5 sec, 60°C for 8 sec (-1°C /cycle) and 68°C for 15 sec. Then perform 35 cycles of 96 °C 5 sec, 45°C for 8 sec and 68°C for 15 sec. Finish with 72°C for 3 min	460 bp
ROUND 2	PVO3;CCADMCBTTTTGYCKYARRCCTTC<br>PVOnstF;AARTGGAAYAAYCAYCARMG	95°C 5 min, then 14 cycles of 96°C for 5 sec, 60°C for 8 sec (-1°C /cycle) and 68°C for 9 sec. Then perform 35 cycles of 96°C 5 sec, 45°C for 8 sec and 68°C for 9 sec. Finish with 72°C for 3 min	260 bp

NOTE: None.

P-017 Lyssaviruses

REFERENCE: **Vazquez-Moron, S. *et al* (2006)** [15]
TARGET: Nucleoprotein
CONTROL: UC2
ENZYME: QIAGEN Fast Cycling PCR Kit (Cat. No. 203745)

	PRIMERS	PROTOCOL	AMPLICON
ROUND 1	GRAB1F;AARATNGTRGARCAYCACAC<br>GRAB1R;GCRTTSGANGARTAAGGAGA	95°C for 5 min, fthen 40 cycles of 96°C for 5 sec, 53°C for 8 sec and 68°C for 12 sec. Finish with 72°C for 3 min	373 bp
ROUND 2	GRAB2F;AARATGTGYGCIAAYTGGAG<br>GRAB2R; TCYTGHCCIGGCTCRAACAT	95°C 5 min, then 40 cycles of 96°C for 5 sec, 53°C for 8 sec and 68°C for 9 sec. Finish with 72°C for 3 min	259 bp

NOTE: None

P-018 Seadornaviruses

REFERENCE: **Unpublished. Designed at CII.**
TARGET: Segment 1
CONTROLS: UC1
ENZYME: QIAGEN Fast Cycling PCR Kit (Cat. No. 203745)

	PRIMERS	PROTOCOL	AMPLICON
ROUND 1	USead-F1;WTSAARGGDGGIACITCITCIGC	95°C for 5 min, then 40 cycles of 96°C for 5 sec, 55°C for 8 sec and 68°C for 24 sec. Finish with a final extension of 72°C for 4 min	756 bp
	USead-R1;GTWGTYAAYTCRCCIGAYTTDACICC		
ROUND 2	USead-F2;GGIACITCITCIGCIAGTAGIAC	Same for both rounds	747 bp
	USead-R1 same as round 1		

NOTE: Seadornaviruses have a dsRNA genome. For greatest efficiency we therefore recommend denaturing the dsRNA prior to reverse-transcription (even though assays for other dsRNA viruses such as orbiviruses do still seem to work without RNA denaturation. See Palacios *et al*, J.Clin.Microbiol 2011). Denature at 95 °C for 5 min and snap cool on ice.

P-019 Alphaviruses

REFERENCE: **Sanchez-Seco, M.P. *et al* (2001)** [16]
TARGET: NSP4
CONTROLS: UC1
ENZYME: QIAGEN Fast Cycling PCR Kit (Cat. No. 203745)

	PRIMERS	PROTOCOL	AMPLICON
ROUND 1	Alpha1F;GAYGCITAYYTIGAYATGGTIGAIGG	95°C 5 min, then 40 cycles of 96°C for 5 sec, 52°C for 8 sec and 68°C for 15 sec. Finish with 72°C for 3 min	481 bp
	Alpha1R;KYTCYTCIGTRTGYTTIGTICCIGG		
ROUND 2	Alpha2F;GIAAYTGYAAYGTIACICARATG	95°C 5 min, then 40 cycles of 96°C for 5 sec, 52°C for 8 sec and 68°C for 8 sec. Finish with 72°C for 2 min	195 bp
	Alpha2R;GCRAAIARIGCIGCIGCYTYIGGICC		

NOTES: The first primer pair will often work well enough on their own, but there is a nested set included to increase sensitivity where required (recommended). Please note that these primers can, on occasion, produce a non-specific product (host) of the expected size. All positives must be confirmed by sequencing.

P-020 Hantaviruses

REFERENCE: **Klempa, B. *et al* (2006)** [17]
TARGET: L-Segment
CONTROLS: Hantavirus cDNA. Not included in either UC1 or 2
ENZYME: QIAGEN Fast Cycling PCR Kit (Cat. No. 203745)

	PRIMERS	PROTOCOL	AMPLICON
ROUND 1	HAN-L-F1;ATGTAYGTBAGTGCWGATGC	95°C 5 min, then 7 cycles of 96°C for 5 sec, 60°C (-1°C per cycle) for 8 sec and 68°C for 18 sec. Then 35 cycles of 96°C for 5 sec, 53°C for 8 sec and 68°C for 18 sec. Finish with 72°C for 4 min	-
	HAN-L-R1;AACCADTCWGTYCCRTCATC		

ROUND 2	HAN-L-F2;TGCWGATGCHACIAARTGGTC<br>HAN-L-R2;GCRTCRTCWGARTGRTGDGCAA	95°C 5 min, then 10 cycles of 96°C for 5 sec, 65°C (-1°C per cycle) for 8 sec and 68°C for 18 sec. Then 35 cycles of 96°C for 5 sec, 55°C for 8 sec and 68°C for 18 sec. Finish with 72°C for 4 min	~412 bp

NOTES: We have used this assay to find several new hantaviruses in different species, including dolphins and bats.

P-021 Phleboviruses

REFERENCE: **Sanchez-Seco, M. P. *et al* (2003)** [18]
TARGET: L-Segment
CONTROLS: UC2
ENZYME: QIAGEN Fast Cycling PCR Kit (Cat. No. 203745)

	PRIMERS	PROTOCOL	AMPLICON
ROUND 1	NPhlebo1POS;ATGGARGGITTTGTIWSICIICC<br>NPhlebo1NEG;AARTTRCTIGWIGCYTTIARIGTIGC	95°C 5 min, then 40 cycles of 96°C for 5 sec, 45°C for 8 sec and 68°C for 18 sec. Finish with 72°C for 3 min	553 bp
ROUND 2	NPhlebo2POS;WTICCIAAICCIYMSAARATG<br>NPhlebo2NEG;TCYTCYTTRTTYTTRARRTARCC	95°C 5 min, then 40 cycles of 96°C for 5 sec, 45°C for 8 sec and 68°C for 9 sec. Finish with 72°C for 2 min	244 bp

NOTES: None

P-022 Orthobunyavirus

REFERENCE: **Briese, T. *et al.* (2007)** [19]
TARGET: S segment
CONTROLS: UC2
ENZYME: QIAGEN Fast Cycling PCR Kit (Cat. No. 203745)

	PRIMERS	PROTOCOL	AMPLICON
ROUND 1	BUNS-5-U-6;*CGGCGCC*AGTAGTGTACTCCAC<br>BUNS-3-L947;*GCGGCC*AGTAGTGTGCTCCAC	95°C 5 min, then 45 cycles of 96°C for 5 sec, 48°C for 8 sec and 68°C for 30 sec. Finish with 72°C for 3 min	~930 bp

NOTES: This paper contains primer sets for all three segments of the bunyaviruses. It is recommended that investigators use at least two of these when screening for orthobunyaviruses (see also M below).

P-023 Orthobunyavirus

REFERENCE: **Briese, T. *et al.* (2007)** [19]
TARGET: M segment
CONTROLS: UC2

ENZYME: QIAGEN Fast Cycling PCR Kit (Cat. No. 203745)

	PRIMERS	PROTOCOL	AMPLICON
ROUND 1	M3560-F;TCNAARGGHTGYGGNAATGT	95°C 5 min, then 45 cycles of 96°C for 5 sec, 48°C for 8 sec and 68°C for 30 sec. Finish with 72°C for 3 min	~898 bp
	BUN-S3-R;*CGCGCC*AGTAGTGTGCTACC		

NOTES: This paper contains primer sets for all three segments of the bunyaviruses. It is recommended that investigators use at least two of these when screening for orthobunyaviruses (see also S above).

P-024 Caliciviruses

REFERENCE: **Reid, S.M. *et al.* (1999)** [20]
TARGET: Capsid-coding region
CONTROLS: Calicivirus cDNA
ENZYME: QIAGEN Fast Cycling PCR Kit (Cat. No. 203745)

	PRIMERS	PROTOCOL	AMPLICON
ROUND 1	1F;GTGAGGTGTTTGAGAATTAG	95°C for 5 mins, then 35 cycles of 96°C for 5 sec, 55°C for 8 sec, 68°C for 24 sec. Finish with 72°C for 3 min	768bp
	1R;ACATCAATTCCGCCAGACCA		

NOTES: None

P-025 Influenza A viruses

REFERENCE: **Anthony, S.J. et al (2012)** [21]
TARGET: Matrix
CONTROLS: UC2
ENZYME: AmpliTaq Gold 360 Master Mix (Applied Biosystems Cat. No. 439881)

	PRIMERS	PROTOCOL	AMPLICON
ROUND 1	FLUAV-MU44;GTCTTCTAACCGAGGTCGAAACG	95°C for 10 mins, then 14 cycles of 95°C for 30 sec, 65°C (-1°C/cycle) for 30 sec, 72°C for 1 min. Then 35 cycles of 95°C for 30 sec, 50°C for 30 sec, 72°C for 1 min. Final extension of 72°C for 7 min	~240 bp
	FLUAV-M-L287;GCATTTTGGACAAAGCGTCTACG		

NOTE: None.

P-026 Influenza A

REFERENCE: **Liang, E. Unpublished. Developed at CII.**
TARGET: PB1
CONTROL: Influenza A cDNA. Not included in either UC1 or 2
ENZYME: QIAGEN Fast Cycling PCR Kit (Cat. No. 203745)

	PRIMERS	PROTOCOL	AMPLICON
ROUND 1	FLUAPB1-F;ATGATGATGGGNATGTTYAAYATG<br>FLUAPB1-R;GCNGGNCCNAKDTCRYTRTTDATCAT	95°C for 5 min, then 14 cycles of 96°C for 5 sec, 65°C for 8 sec (-1°C /cycle) and 68°C for 15 sec. Then perform 35 cycles of 96°C for 5 sec, 50°C for 8 sec and 68°C for 15 sec. Finish with 72°C for 4 min	407 bp
ROUND 2	FLUAPB1-NF;GATGGGNATGTTYAAYATGYTDAGYAC<br>FLUAPB1-R Same reverse primer as Round 1	Same protocol for both rounds	402 bp

NOTES: This assay was designed to be more degenerate than P-025 above. It was developed to capture the highly diverse bat influenza viruses (recently discovered) in addition to all 'classic' influenza A viruses. While a nested assay is presented here, we have to date not observed any increased sensitivity from the nested round.

P-027 Simian Foamy Virus

REFERENCE: **Goldberg, T.L. *et al* (2009)** [22]
TARGET: RdRp
CONTROLS: UC2
ENZYME: QIAGEN Fast Cycling PCR Kit (Cat. No. 203745)

	PRIMERS	PROTOCOL	AMPLICON
ROUND 1	SIF2: TAGCWGAYAARCTTGCCACCCAAGG<br>SIR1: GTCGTTTWATITCACTATTTTTCCTTTCCAC	95°C 5 min, then 10 cycles of 96°C for 5 sec, 65°C (-1°C /cycle) for 8 sec and 68°C for 24 sec. Then 35 cycles of 96°C for 5 sec, 55°C for 8 sec and 68°C for 24 sec. Finish with 72°C for 3 min	729 bp
ROUND 2	SIF3: CCAARCCTGGATGCAGAGYTGGATCA<br>SIR3: ACTTTGGGGRTGRTAAGGAGTACTG	95°C 5 min, then 10 cycles of 96°C for 5 sec, 65°C (-1°C /cycle) for 8 sec and 68°C for 21 sec. Then 35 cycles of 96°C for 5 sec, 55°C for 8 sec and 68°C for 21 sec. Finish with 72°C for 3 min	632 bp

NOTES: None

P-028 Simian Foamy Virus

REFERENCE: **Goldberg, T.L. *et al* (2009)** [22]
TARGET: LTR
CONTROLS: UC2

ENZYME: QIAGEN Fast Cycling PCR Kit (Cat. No. 203745)

	PRIMERS	PROTOCOL	AMPLICON
ROUND 1	FVRU5F1: CACTRCTCGCTGCGYCGAGAGT	95°C 5 min, then 10 cycles of 96°C for 5 sec, 65°C (-1°C /cycle) for 8 sec and 68°C for 12 sec. Then 35 cycles of 96°C for 5 sec, 55°C for 8 sec and 68°C for 12 sec Finish with 72°C for 3 min	364 bp
	FVRU5R1: CCCGACTTATATTCGAGCCCCAC		
ROUND 2	FVRU5F2: GAGWCTCTCCAGGYTTGGTAAGA	Same for both rounds	314 bp
	FVRU5R2: CACGTTGGGCGCCAATTGT		

NOTES: None

P-029 Lentivirus

REFERENCE: **Clewley, J.P. *et al* (1998)** [23]
TARGET: RdRp
CONTROLS: UC2
ENZYME: QIAGEN Fast Cycling PCR Kit (Cat. No. 203745)

	PRIMERS	PROTOCOL	AMPLICON
ROUND 1	DR1;TRCAYACAGGRGCWGAYGA	95°C 5 min, then 14 cycles of 96°C for 5 sec, 65°C (-1°C /cycle) for 8 sec and 68°C for 24 sec. Then 35 cycles of 96°C for 5 sec, 50°C for 8 sec and 68°C for 24 sec. Finish with 72°C for 4 min	785 bp
	DR2;AIADRTCATCCATRTAYTG		
ROUND 2	DR4;GGIATWCCICAYCCDGCAGG	95°C 5 min, then 14 cycles of 96°C for 5 sec, 65°C (-1°C /cycle) for 8 sec and 68°C for 9 sec. Then 35 cycles of 96°C for 5 sec, 50°C for 8 sec and 68°C for 9 sec. Finish with 72°C for 3 min	194 bp
	DR5;GGIGAYCCYTTCCAYCCYTGHGG		

NOTES: None

P-030 Picobirnaviruses

REFERENCE: **Anthony, S; Unpublished. Designed at CII.**
TARGET: Segment 2
CONTROL: Picobirnavirus cDNA. Not included in either UC1 or 2.
ENZYME: QIAGEN Fast Cycling PCR Kit (Cat. No. 203745)

	PRIMERS	PROTOCOL	AMPLICON
ROUND 1	PicoF3;GTDRTDTGGATGTTYCC	95°C 5 min, then 40 cycles of 96°C for 5 sec, 48°C for 8 sec and 68°C for 26 sec. Finish with 72°C for 4 min	~800bp
	PicoF5;GTHTGGATGTWYCCIATG		
	PicoR5;GGRTGRTAYTTVCARTTYTC		
	PicoR8;GGRTBRTCHACACARTTYTC		

NOTES: Use all 4 primers. This assay was designed using a substantial picobirnavirus diversity observed in the macaques sampled for this study. It is able to detect both genotype 1 and genotype 2 viruses.

DNA Virus Protocols

P-031 Herpesviruses

REFERENCE: **Van DeVanter, D.R. *et al* (1996)** [24]
TARGET: Polymerase
CONTROL: UC2
ENZYME: QIAGEN Fast Cycling PCR Kit (Cat. No. 203745)

	PRIMERS	PROTOCOL	AMPLICON
ROUND 1	DFA;GAYTTYGCNAGYYTNTAYCC	95°C 5 min, then 45 cycles of 96°C for 5 sec, 46°C for 8 sec and 68°C for 12 sec. Finish with 72°C for 2 min	-
	KG1;GTCTTGCTCACCAGNTCNACNCCYTT		
	ILK;TCCTGGACAAGCAGCARNYSGCNMTNAA		
ROUND 2	TGV;TGTAACTCGGTGTAYGGNTTYACNGGNGT	Same protocol for both rounds	215-315 bp
	IYG;CACAGAGTCCGTRTCNCCRTADAT		

NOTES: Very well tested assay, and shown to work well on clinical samples. This assay has a bias for gammaherpesviruses. It will still detect alpha and beta herpesviruses, but if there is a co-infection between a gamma and either alpha or beta, it will preferentially amplify the gamma (so investigators should always consider cloning to identify co-infections). **Important: this assay requires the addition of dimethyl sulphoxide (DMSO) to final dilution of 5% total volume** (e.g. 1μl in a 20μl reaction).

P-032 Herpesviruses

REFERENCE: **Chmielewicz, B. *et al* (2001)** [25]
TARGET: Terminase
CONTROL: UC2
ENZYME: QIAGEN Fast Cycling PCR Kit (Cat. No. 203745)

	PRIMERS	PROTOCOL	AMPLICON
ROUND 1	TS-TERM_707s;TTGTGGACGAGRSIMAYTTYAT	95°C 5 min, then 45 cycles of 96°C for 5 sec, 46°C for 8 sec and 68°C for 12 sec. Finish with 72°C for 2 min	519bp
	TS-TERM_707as;ACAGCCACGCCNGTICCIGAIGC		
ROUND 2	TS-TERM_708s;GCAAGATCATNTTYRTITCITC	Same protocol for both rounds	419bp
	TS-TERM_708as;TGTTGGTCGTRWAIGCIGGRT		

NOTES: This PCR produces a larger product than P-031. While this can have advantages for sequencing and characterization of new strains, there are fewer sequences for this gene in the databases for comparison. **Important: this assay requires the addition of dimethyl sulphoxide (DMSO) to final dilution of 5% total volume (e.g. 1µl in a 20µl reaction)**.

P-033 Bocaviruses

REFERENCE: **Kapoor, A. *et al* (2010)** [26]
TARGET: NS1
CONTROL: UC1
ENZYME: QIAGEN Fast Cycling PCR Kit (Cat. No. 203745)

	PRIMERS	PROTOCOL	AMPLICON
ROUND 1	panBOV-F1;TAATGCAYCARGAYTGGGTIGANCC	95°C 5 min, then 6 cycles of: 96°C for 5 sec, 61°C for 8 sec and 68°C for 9 sec. Then 35 cycles of: 96°C for 5 sec, 59°C for 8 ec and 68°C for 9 sec. Finish with 72°C for 2 min	293 bp
	panBOV-R1;GTACAGTCRTAYTCRTTRAARCACCA		
ROUND 2	panBOV-F2;GCAYCARGAYTGGGTIGANCCWGC	95°C 5 min, then 6 cycles of: 96°C for 5 sec, 60°C for 8 sec and 68°C for 9 sec. Then 35 cycles of: 96°C for 5 sec, 58°C for 8 sec and 68°C for 9 sec. Finish with 72°C for 2 min	290 bp
	panBOV-R1 Same reverse as round 1		

NOTE: None

P-034 Poxviruses

REFERENCE: **Bracht, A.J. *et al* (2006)** [27]
TARGET: Polymerase
CONTROL: UC2
ENZYME: QIAGEN Fast Cycling PCR Kit (Cat. No. 203745)

	PRIMERS	PROTOCOL	AMPLICON
ROUND 1	FP-DNApol;ATACAGAGCTAGTACITTAATAAAAG	95°C 5 min, then 45 cycles of 96°C for 5 sec, 45°C for 8 sec and 68°C for 15 sec. Finish with 72°C for 2 min	~543 bp
	RP-DNApol;CTATTTTTAAATCCCATTAAACC		

NOTES: This is a paper describing several poxvirus PCRs. The protocol described here is the polymerase assay. The topoisomerase assay is described below (P-035). I**mportant: this assay requires the addition of dimethyl sulphoxide (DMSO) to final dilution of 5% total volume (e.g. 1µl in a 20µl reaction)**.

P-035 Poxviruses

REFERENCE: **Bracht, AJ et al (2006)** [27]
TARGET: Topoisomerase 1
CONTROL: UC2
ENZYME: QIAGEN Fast Cycling PCR Kit (Cat. No. 203745)

	PRIMERS	PROTOCOL	AMPLICON
ROUND 1	FP-DNAtopo;TAATGGAAACAAGTTTTTTTAT<br>RP-DNAtopo;CCAAAAATTATATAAAAACG	95°C 5 min, then 45 cycles of 96°C for 5 sec, 45°C for 8 sec and 68°C for 10 sec. Finish with 72°C for 2 min	~344 bp

NOTES: This is a paper describing several poxvirus PCRs. The protocol described here is the topoisomerase assay. The polymerase assay is described above (P-034). I**mportant: this assay requires the addition of dimethyl sulphoxide (DMSO) to final dilution of 5% total volume (e.g. 1μl in a 20μl reaction).**
.

P-036 Orthopoxviruses

REFERENCE: **Nitsche, A. *et al* (2004)** [28]
TARGET: rpo18 subunit of the RNA polymerase
CONTROL: UC2
ENZYME: QIAGEN Fast Cycling PCR Kit (Cat. No. 203745)

	PRIMERS	PROTOCOL	AMPLICON
ROUND 1	rpo OPV-F1;CTGTAGTTATAAACGTTCCGTGTG<br>rpo OPV-R1;TTATCATACGCATTACCATTTCGA	95°C 5 min, then 40 cycles of 96°C for 5 sec, 54°C for 8 sec and 68°C for 8 sec. Finish with 72°C for 2 min	204 bp

NOTES: Consensus assay for the orthopox genus, rather than all poxviruses. It is not as broadly reactive as the pan-pox assays, but is well tested on different orthopox species.

P-037 Parapoxvirus

REFERENCE: **Inoshima, Y. *et al* (2000)** [29]
TARGET: B2L
CONTROL: UC2
ENZYME: QIAGEN Fast Cycling PCR Kit (Cat. No. 203745)

	PRIMERS	PROTOCOL	AMPLICON
ROUND 1	PPP-1;GTCGTCCACGATGAGCAGCT	95°C 5 min, then 5 cycles of 96°C for 5 sec, 50°C for 8 sec and 68°C for 18 sec. Then 35 cycles of 96°C for 5 sec, 55°C for 8 sec and 68°C for 18 sec. Finish with 72°C for 4 min	594 bp
	PPP-4;TACGTGGGAAGCGCCTCGCT		
ROUND 2	PPP-3;GCGAGTCCGAGAAGAATACG	95°C 5 min, then 5 cycles of 96°C for 5 sec, 50°C for 8 sec and 68°C for 8 sec. Then 35 cycles of 96°C for 5 sec, 55°C for 8 sec and 68°C for 9 sec. Finish with 72°C for 3 min	235 bp
	PPP-4 Same as round 1		

NOTES: Consensus assay for the parapox genus, rather than all poxviruses. It is not as broadly reactive as the pan-pox assays, but is well tested on different parapox species.

P-038 Papillomaviruses

REFERENCE: **Forslund, O. *et al* (1999)** [30]
TARGET: L1
CONTROL: UC2
ENZYME: QIAGEN Fast Cycling PCR Kit (Cat. No. 203745)

	PRIMERS	PROTOCOL	AMPLICON
ROUND 1	FAP/59;TAACWGTIGGICAYCCWTATT	95°C 5 min, then 40 cycles of 96°C for 5 sec, 50°C for 8 sec and 68°C for 15 sec. Finish with 72°C for 2 min	476 bp
	FAP/64;CCWATATCWVHCATITCICCATC		

NOTES: This assay was developed to detect human papilloma viruses. It has been used to detect some animal viruses too (eg: Antonsson, A; 2002; Journal of Virology, 76: 12537–12542) but there are also reports that show these primers missed other novel animal papillomas. (eg: Rai et al, 2011, Vet Micro 147: 416-419).

P-039 Adenoviruses

REFERENCE: **Wellehan, J. *et al* (2004)** [31]
TARGET: Polymerase
CONTROL: UC2
ENZYME: AmpliTaq Gold 360 Master Mix (Applied Biosystems Cat. No. 439881)

	PRIMERS	PROTOCOL	AMPLICON
ROUND 1	FLTR;TIMGNGGIGGIMGNTGYTAYCC	95°C for 10 mins, then 14 cycles of 95°C for 30 sec, 65°C (-1°C/cycle) for 30 sec, 72°C for 1 min. Then 35 cycles of 95°C for 30 sec, 50°C for 30 sec, 72°C for 1 min. Finish with 72°C for 7 min	~550 bp
	RTR;GTDGCRAAISHICCRTABARIGMRTT		
ROUND 2	FNR;GTITWYGAYATHTGYGGHATGTAYGC	Protocol same for both rounds	~320 bp
	RNR;CCAICCBCDRTTRTGIARIGTRA		

NOTES: This assay has been widely used to detect novel adenoviruses in animals, including many bat, bird and reptilian adenoviruses, from all four genera. They have even been used to identify a putative new genus in the family. Please note: we have tested this protocol with multiple enzymes, and only seems to work reliably with AmpliTaq Gold.

P-040 Polyomaviruses

REFERENCE: **Johne, R (2005)[32]**
TARGET: VP1
CONTROL: UC2
ENZYME: QIAGEN Fast Cycling PCR Kit (Cat. No. 203745)

	PRIMERS	PROTOCOL	AMPLICON
ROUND 1	VP1/lf;CCAGACCCAACTARRAATGARAA	95°C 5 min, then 45 cycles of 96°C for 5 sec, 46°C for 8 sec and 68°C for 30 sec. Finish with 72°C for 3 min	829-1137 bp
	VP1/1r;AACAAGAGACACAAATNTTTCCNCC		
ROUND 2	VP1/2f;ATGAAAATGGGGTTGGCCCNCTNTGYAARG	95°C 5 min, then 45 cycles of 96°C for 5 sec, 56°C for 8 sec and 68°C for 30 sec. Finish with 72°C for 3 min	249-273 bp
	VP1/2r;CCCTCATAAACCCGAACYTCYTCHACYTG		

NOTES: An assay originally used to identify novel polyoma in chimps. But was designed to be broadly reactive, and we have used it successfully to identify several novel polyomaviruses in diverse species (including dolphins).

P-041 Polyomaviruses

REFERENCE: **Johne, R. *et al* (2005)** [32]
TARGET: VP3
CONTROL: Any polyomavirus DNA. Not included in either UC1 or 2.
ENZYME: QIAGEN Fast Cycling PCR Kit (Cat. No. 203745)

	PRIMERS	PROTOCOL	AMPLICON
ROUND 1	VP3/lf;CTCCAGGAGGTGCAMABCAAMG	95°C 5 min, then 14 cycles of 96°C for 5 sec, 65°C (-1°C /cycle) for 8 sec and 68°C for 21 sec. Then 35 cycles of 96°C for 5 sec, 50°C for 8 sec and 68°C for 21 sec. Finish with 72°C for 3 min	~560-630 bp
	VP3/1r;TGCAATTCCAGAGGTTCNCCNCCNAC		
ROUND 2	VP3/2f;ACTGGATGCTGCCTYTAMTTYTAGG	95°C 5 min, then 14 cycles of 96°C for 5 sec, 65°C (-1°C /cycle) for 8 sec and 68°C for 15 sec. Then 35 cycles of 96°C for 5 sec, 50°C for 8 sec and 68°C for 15 sec. Finish with 72°C for 3 min	~390-460 bp
	VP3/2r;TCAGTTTTTACAGTTACWGCYTCCCACAT		

NOTES: An assay originally used to identify novel polyoma in chimps. But was designed to be broadly reactive, and we have used it successfully to identify several novel polyomaviruses in diverse species (including dolphins).

Supplementary References

1 Atkins, A. *et al.* Characterization of an outbreak of astroviral diarrhea in a group of cheetahs (Acinonyx jubatus). *Veterinary microbiology* **136**, 160-165, doi:10.1016/j.vetmic.2008.10.035 (2009).

2 Chu, D. K., Poon, L. L., Guan, Y. & Peiris, J. S. Novel astroviruses in insectivorous bats. *Journal of virology* **82**, 9107-9114, doi:10.1128/JVI.00857-08 (2008).
3 Lozano, M. E. *et al.* Characterization of arenaviruses using a family-specific primer set for RT-PCR amplification and RFLP analysis - Its potential use for detection of uncharacterized arenaviruses. *Virus Res* **49**, 79-89, doi:Doi 10.1016/S0168-1702(97)01458-5 (1997).
4 Quan, P. L. *et al.* Identification of a severe acute respiratory syndrome coronavirus-like virus in a leaf-nosed bat in Nigeria. *mBio* **1**, doi:10.1128/mBio.00208-10 (2010).
5 Watanabe, S. *et al.* Bat Coronaviruses and Experimental Infection of Bats, the Philippines. *Emerging infectious diseases* **16**, 1217-1223, doi:Doi 10.3201/Eid1608.100208 (2010).
6 Zhai, J. H. *et al.* Rapid molecular strategy for filovirus detection and characterization. *Journal of clinical microbiology* **45**, 224-226, doi:Doi 10.1128/Jcm.01893-06 (2007).
7 Moureau, G. *et al.* A Real-Time RT-PCR Method for the Universal Detection and Identification of Flaviviruses. *Vector Borne Zoonotic Dis* **7**, 467-478 (2007).
8 Sanchez-Seco, M. P. *et al.* Generic RT-nested-PCR for detection of flaviviruses using degenerated primers and internal control followed by sequencing for specific identification. *Journal of virological methods* **126**, 101-109, doi:Doi 10.1016/J.Jviromet.2005.01.025 (2005).
9 Woo, P. C. Y. *et al.* Complete Genome Sequence of a Novel Picornavirus, Canine Picornavirus, Discovered in Dogs. *Journal of virology* **86**, 3402-3403, doi:Doi 10.1128/Jvi.07228-11 (2012).
10 Nix, W. A., Oberste, M. S. & Pallansch, M. A. Sensitive, seminested PCR amplification of VP1 sequences for direct identification of all enterovirus serotypes from original clinical specimens. *Journal of clinical microbiology* **44**, 2698-2704, doi:Doi 10.1128/Jcm.00542-06 (2006).
11 Nollens, H. H. *et al.* New recognition of Enterovirus infections in bottlenose dolphins (Tursiops truncatus). *Veterinary microbiology* **139**, 170-175, doi:10.1016/j.vetmic.2009.05.010 (2009).
12 Tong, S., Chern, S. W., Li, Y., Pallansch, M. A. & Anderson, L. J. Sensitive and broadly reactive reverse transcription-PCR assays to detect novel paramyxoviruses. *Journal of clinical microbiology* **46**, 2652-2658, doi:10.1128/JCM.00192-08 (2008).
13 Feldman, K. S. *et al.* Design and evaluation of consensus PCR assays for henipaviruses. *Journal of virological methods* **161**, 52-57, doi:Doi 10.1016/J.Jviromet.2009.05.014 (2009).
14 Bourhy, H., Cowley, J. A., Larrous, F., Holmes, E. C. & Walker, P. J. Phylogenetic relationships among rhabdoviruses inferred using the L polymerase gene. *J Gen Virol* **86**, 2849-2858, doi:Doi 10.1099/Vir.0.81128-0 (2005).
15 Vazquez-Moron, S., Avellon, A. & Echevarria, J. E. RT-PCR for detection of all seven genotypes of Lyssavirus genus. *Journal of virological methods* **135**, 281-287, doi:Doi 10.1016/J.Jviromet.2006.03.008 (2006).

16 Sanchez-Seco, M. P., Rosario, D., Quiroz, E., Guzman, G. & Tenorio, A. A generic nested-RT-PCR followed by sequencing for detection and identification of members of the alphavirus genus. *Journal of virological methods* **95**, 153-161, doi:Doi 10.1016/S0166-0934(01)00306-8 (2001).
17 Klempa, B. *et al.* Hantavirus in African Wood Mouse, Guinea. *Emerging infectious diseases* **12**, 838-840 (2006).
18 Sanchez-Seco, M. P. *et al.* Detection and identification of Toscana and other phleboviruses by RT-nested-PCR assays with degenerated primers. *J Med Virol* **71**, 140-149, doi:Doi 10.1002/Jmv.10465 (2003).
19 Briese, T., Kapoor, V. & Lipkin, W. I. Natural M-segment reassortment in Potosi and Main Drain viruses:implications for the evolution of orthobunyaviruses. *Archives of virology* **152**, 2237–2247 (2007).
20 Reid, S. M. *et al.* Development of a reverse transcription polymerase chain reaction procedure for the detection of marine caliciviruses with potential application for nucleotide sequencing. *Journal of virological methods* **82**, 99-107, doi:Doi 10.1016/S0166-0934(99)00088-9 (1999).
21 Anthony, S. J. *et al.* Emergence of fatal avian influenza in New England harbor seals. *mBio* **3**, e00166-00112, doi:10.1128/mBio.00166-12 (2012).
22 Goldberg, T. L. *et al.* Coinfection of Ugandan Red Colobus (Procolobus [Piliocolobus] rufomitratus tephrosceles) with Novel, Divergent Delta-, Lenti-, and Spumaretroviruses. *Journal of virology* **83**, 11318-11329, doi:Doi 10.1128/Jvi.02616-08 (2009).
23 Clewley, J. P., Lewis, J. C., Brown, D. W. & Gadsby, E. L. A novel simian immunodeficiency virus (SIVdrl) pol sequence from the drill monkey, Mandrillus leucophaeus. *J Virol* **72**, 10305-10309 (1998).
24 VanDevanter, D. R. *et al.* Detection and analysis of diverse herpesviral species by consensus primer PCR. *Journal of clinical microbiology* **34**, 1666-1671 (1996).
25 Chmielewicz, B., Goltz, M. & Ehlers, B. Detection and multigenic characterization of a novel gammaherpesvirus in goats. *Virus Res* **75**, 87-94, doi:Doi 10.1016/S0168-1702(00)00252-5 (2001).
26 Kapoor, A. *et al.* Identification and characterization of a new bocavirus species in gorillas. *Plos One* **5**, e11948, doi:10.1371/journal.pone.0011948 (2010).
27 Bracht, A. J. *et al.* Genetic identification of novel poxviruses of cetaceans and pinnipeds. *Archives of virology* **151**, 423-438, doi:Doi 10.1007/S00705-005-0679-6 (2006).
28 Nitsche, A., Ellerbrok, H. & Pauli, G. Detection of orthopoxvirus DNA by real-time PCR and identification of variola virus DNA by melting analysis. *J Clin Microbiol* **42**, 1207-1213 (2004).
29 Inoshima, Y., Morooka, A. & Sentsui, H. Detection and diagnosis of parapoxvirus by the polymerase chain reaction. *J Virol Methods* **84**, 201-208 (2000).
30 Forslund, O., Antonsson, A., Nordin, P., Stenquist, B. & Hansson, B. G. A broad range of human papillomavirus types detected with a general PCR method suitable for analysis of cutaneous tumours and normal skin. *J Gen Virol* **80**, 2437-2443 (1999).

31 Wellehan, J. F. *et al.* Detection and analysis of six lizard adenoviruses by consensus primer PCR provides further evidence of a reptilian origin for the atadenoviruses. *Journal of virology* **78**, 13366-13369, doi:10.1128/JVI.78.23.13366-13369.2004 (2004).
32 Johne, R., Enderlein, D., Nieper, H. & Muller, H. Novel polyomavirus detected in the feces of a chimpanzee by nested broad-spectrum PCR. *Journal of virology* **79**, 3883-3887, doi:10.1128/JVI.79.6.3883-3887.2005 (2005).
